# Delivery of miR-15b-5p via magnetic nanoparticle-enhanced bone marrow mesenchymal stem cell-derived extracellular vesicles mitigates diabetic osteoporosis by targeting GFAP

**DOI:** 10.1007/s10565-024-09877-2

**Published:** 2024-07-05

**Authors:** Chen Xu, Zhaodong Wang, Yajun Liu, Keyou Duan, Jianzhong Guan

**Affiliations:** 1 Department of Orthopedics, Bengbu Medical University Affiliated to First Hospital, Anhui Province Key Laboratory of Tissue Transplantation (Bengbu Medical College), 2600 Donghai Avenue, No. 287, Changhuai Road, Longzihu District, Bengbu, 233000 Anhui Province China; 2 Anhui Province Key Laboratory of Tissue Transplantation (Bengbu Medical College), 2600 Donghai Avenue, Bengbu, 233030 China

**Keywords:** Magnetic nanoparticle, Bone marrow mesenchymal stem cell, Extracellular vesicle, miR-15b-5p, GFAP, Diabetic osteoporosis, Osteoclast differentiation

## Abstract

Diabetic osteoporosis (DO) presents significant clinical challenges. This study aimed to investigate the potential of magnetic nanoparticle-enhanced extracellular vesicles (GMNP_E_-EVs) derived from bone marrow mesenchymal stem cells (BMSCs) to deliver miR-15b-5p, thereby targeting and downregulating glial fibrillary acidic protein (GFAP) expression in rat DO models. Data was sourced from DO-related RNA-seq datasets combined with GEO and GeneCards databases. Rat primary BMSCs, bone marrow-derived macrophages (BMMs), and osteoclasts were isolated and cultured. EVs were separated, and GMNP_E_ targeting EVs were synthesized. Bioinformatic analysis revealed a high GFAP expression in DO-related RNA-seq and GSE26168 datasets for disease models. Experimental results confirmed elevated GFAP in rat DO bone tissues, promoting osteoclast differentiation. miR-15b-5p was identified as a GFAP inhibitor, but was significantly downregulated in DO and enriched in BMSC-derived EVs. In vitro experiments showed that GMNP_E_-EVs could transfer miR-15b-5p to osteoclasts, downregulating GFAP and inhibiting osteoclast differentiation. In vivo tests confirmed the therapeutic potential of this approach in alleviating rat DO. Collectively, GMNP_E_-EVs can effectively deliver miR-15b-5p to osteoclasts, downregulating GFAP expression, and hence, offering a therapeutic strategy for rat DO.

**Supplementary Information:**

The online version contains supplementary material available at 10.1007/s10565-024-09877-2.

## Introduction

The rising prevalence of diabetes mellitus worldwide has precipitated an increase in research interest in its complications, among which diabetic osteoporosis (DO) has emerged as a noteworthy concern (Rasmussen and Vestergaard 2022). The incidence of DO is increasing due to the rising number of diabetes patients, making it a growing public health concern (Duru et al. 2018). Unlike primary osteoporosis, which is traditionally characterized by a significant decrease in bone mineral density, DO presents a unique challenge to clinicians and researchers alike. It is often not accompanied by a substantial reduction in bone density, making its diagnosis and management more complex (Brown 2021). The potential of emerging technologies and therapeutic agents, specifically designed to target the molecular pathways involved in DO, offers a promising avenue for mitigating the impact of this condition.

In recent years, the integration of nanotechnology and regenerative medicine has instilled new hope in the treatment of skeletal diseases (Jeong et al. 2022). The utilization of technology involving polyethylene glycol-modified magnetic nanoparticles (GMNPs) loaded into extracellular vesicles (EVs) has garnered interest in the treatment of osteoporosis (Yong and Logan 2021). As a natural biological delivery system, EVs can effectively deliver cargo to target cells, while GMNPs enable precise control and localization (Xu et al. 2024). Using GMNPs to load EVs (GMNP_E_-EVs) allows for precise control over osteoclasts, consequently influencing bone reconstruction (Xu et al. 2023). Existing research highlights the pivotal role of GMNP_E_-EVs in osteogenic differentiation, guiding the molecular interactions linked with osteoclast dynamics and functionalities (Kitaura et al. 2020). This study presents a novel approach to treating osteoporosis (Zhang et al. 2020). According to the bioinformatics analysis conducted in this study, glial fibrillary acidic protein (GFAP) was predicted to play a crucial role in this process by potentially promoting osteoclast differentiation and accelerating disease progression. However, the exact operational schema and precursor mechanisms of GFAP amidst GMNP_E_-EV-driven osteogenic differentiation await elucidation (Zhang et al. 2022). In-depth research on the upstream mechanisms of GFAP is essential to optimize the use of GMNP_E_-EVs in DO treatment.

This study examines the therapeutic effect of using GMNPs loaded with EVs derived from bone marrow mesenchymal stem cells (BMSCs) for delivering miR-15b-5p in the treatment of DO. The research suggests that inhibiting osteoclast differentiation can be achieved by downregulating GFAP using this method. This finding suggests a potential mechanism involving the regulatory role of miR-15b-5p on GFAP, which plays a crucial role in osteoclast differentiation. Hence, the integration of nanotechnology and gene regulation offers a novel approach to the treatment of DO.

## Materials and methods

### Ethics statement

All experimental procedures were approved by the Animal Ethics Committee of Bengbu Medical University Affiliated to First Hospital and conducted in accordance with the Guide for the Care and Use of Laboratory Animals.

### High-throughput transcriptome sample sequencing and data quality control

High-throughput transcriptome sequencing was conducted on three typical rats, and three rats were successfully modeled for DO. Using the Trizol reagent (Thermo, 16,096,020), the total RNA from each specimen was extracted. The concentration, purity, and integrity of the RNA were ascertained utilizing the Qubit®2.0 Fluorometer® (Life Technologies, Q33216) equipped with the Qubit® RNA Assay Kit (Shanghai Bioji Biotechnology Co., Ltd., HKR2106-01, Shanghai, China), the Nanometer Spectrophotometer (IMPLEN), and the RNA Nano 6000 Assay Kit (Agilent, 5067–1511) in the Bioanalyzer 2100 System, respectively. Each sample contained a total RNA content of 3 μg, which was designated as the input material for RNA sample processing. The NEBNext® UltraTM RNA Library Prep Kit (NEB, E7435L, Beijing, China), which was compatible with Illumina® (USA), was employed to produce the subsequent cDNA library. This library's quality was then assessed on the Agilent Bioanalyzer 2100 system. The indexed samples were organized into clusters utilizing the TruSeq PE Cluster Kit v3 cBot HS (Illumina) (PE-401–3001, Illumina) within the cBot Cluster Generation System. Post-cluster generation, the library was prepared on the Illumina-Hiseq 550 platform, resulting in the production of 125 bp/150 bp paired-end reads (Arunachalam et al. 2022; Linkner et al. 2021). The quality of the paired-end reads in the raw sequencing data was inspected using FastQC v0.11.8 software (www.bioinformatics.babraham.ac.uk). The raw data underwent preprocessing through the Cutadapt software 1.18 (www.bioinformatics.babraham.ac.uk), encompassing the removal of Illumina sequencing adaptors and poly(A) tail sequences. Reads with an N content surpassing 5% were eliminated using a Perl script. Those reads boasting a base quality greater than 20 and a coverage of 70% were extracted through the FASTX Toolkit software 0.0.13 (http://hannonlab.cshl.edu/fastx_toolkit/). The BBMap software (https://sourceforge.net/projects/bbmap/) was utilized to rectify paired-end sequences. In the final step, the curated high-quality read fragments were mapped to the reference genome using hisat2 software (version 0.7.12). The DO-related RNA-seq dataset was subsequently procured (Deng et al. 2020; Peng et al. 2019).

### Bioinformatics analysis

The diabetes dataset GSE26168 was obtained by sequencing DO-related RNA-seq data and utilizing the Gene Expression Omnibus (GEO) database (https://www.ncbi.nlm.nih.gov/gds). Differentially expressed genes (DEGs) in two datasets were identified using the "limma" package (Ritchie et al. 2015) of R software and a threshold of P value < 0.05 and |log_10_FC|> 1. A volcano plot was generated using the "ggplot2" package of R software.

To retrieve the DO-related target genes, the GeneCards database (https://www.genecards.org/) was employed, resulting in 1970 genes with a Relevance score > 5. A Venn analysis was conducted on the results of the dataset analysis and the GeneCards database retrieval using the Draw Venn Diagram tool (http://bioinformatics.psb.ugent.be/webtools/Venn/). This analysis led to the identification of the target gene GFAP. Furthermore, protein interactions between GFAP and other genes were predicted through the Genemania website (http://genemania.org/), and protein interactions between target genes and functional genes were analyzed using the String database (https://string-db.org/) (Szklarczyk et al. 2023). Gene ontology (GO) functional annotation and Kyoto Encyclopedia of Genes and Genomes (KEGG) pathway enrichment analysis and visualization were performed on co-expressed genes using the Xiantaozi academic platform (https://www.xiantaozi.com/) (Liu et al. 2021).

To determine the upstream miRNAs of GFAP, the Norwalk database (http://mirwalk.umm.uni-heidelberg.de/interactions/), mipmap database (https://mirmap.ezlab.org/app/) (Vejnar and Zdobnov 2012), and miRBD database (http://www.mirdb.org/) (Chen and Wang 2020) were utilized. The retrieved results were subjected to Venn analysis using the Draw Venn Diagram tool (http://bioinformatics.psb.ugent.be/webtools/Venn/), resulting in the identification of candidate miRNAs.

### DO rat model establishment

Seventy Sprague Wistar rats, aged 7–8 weeks and weighing approximately 180 ± 20 g, were procured from the Vital River Co., Ltd (Beijing, China). These rats were accommodated in an SPF environment, maintaining a temperature of 25 ± 2℃ and a relative humidity between 60–70%. They experienced a 12-h light–dark cycle and were granted unrestricted access to both food and water. Following a week of acclimatization, these rats were ramdonly segregated into a control group (six rats) and a model group (64 rats). In the model group, a type 2 diabetes model was established in 64 rats via streptozotocin injection (60 mg/kg). Random blood glucose concentrations were ascertained, and a level surpassing 16.7 mmol/L confirmed the successful creation of the diabetes model. Concurrently, normal Sprague–Dawley rats were administered a saline solution. An mCT-40 was utilized to scan the femur, and metrics such as bone volume over total volume (BV/TV), trabecular thickness, trabecular number, and bone mineral density (BMD) were deduced via μCT software. After model establishment, every four weeks, parameters such as bone mass volume ratio, trabecular bone thickness, and number of trabecular bones were assessed, and alterations in bone density were observed for both groups. Disparities in BMD and BMC between the groups were discerned, validating the effective development of the DO rat model (Liu et al. 2020).

From the total, 55 therapeutic group rat models were successfully formulated. These were categorized into the GMNP_E_-EVs group, EVs group, and four additional groups—each containing six rats. The four supplementary groups included the overexpression (oe)-NC group (lentiviral oe-NC), the oe-GFAP group (lentiviral oe-GFAP), the sh-NC group (lentiviral sh-NC), and the sh-GFAP group (lentiviral sh-GFAP). Lentiviruses were administered to these groups via the rat tail vein at a functional titer of 5 × 10^6^ TU/mL, with an allocation of 100 μL for each rat for 1 week (Liu et al. 2020).

Forty-eight DO rats were arbitrarily allocated into four distinct groups, with six rats per group. These groups were designated as follows: PBS + oe-NC group (PBS supplemented with lentiviral oe-NC), mimic-NC-GMNP_E_-EVs + oe-NC group (GMNP_E_-EVs derived from BMSCs combined with lentiviral oe-NC), miR-15b-5p-mimic-GMNP_E_-EVs-oe-NC group (GMNP_E_-EVs derived from BMSCs with miR-15b-5p mimic and lentiviral oe-NC), and the miR-15b-5p mimic-GMNP_E_-EVs + oe-GFAP group (GMNP_E_-EVs derived from derived from BMSCs with miR-15b-5p mimic and lentiviral oe-GFAP). Both GMNP_E_-EVs (at a dosage of 400 μL of nanoparticles/kg in PBS) and lentiviral doses (100 μL per individual rat) were delivered to the rats via tail vein injection. To encourage the congregation of GMNP_E_ nanoparticles in the bloodstream at the femoral location, N52 neodymium magnets were used to exert an external magnetic field on the rat's femoral site.

### Detection of gene expression levels by RT-qPCR

The extraction of total RNA was carried out using Trizol Reagent (10,296,010, Thermo Fisher) from Invitrogen. The quality and concentration of RNA were measured using the ND-1000 UV–Vis spectrophotometer (Nanodrop, Thermo Fisher). Reverse transcription was performed utilizing the PrimeScript™ RT-qPCR Kit (RR086A, TaKaRa, Mountain View, California). Real-time quantitative reverse transcription polymerase chain reaction (RT-qPCR) was conducted using the SYBR Premix Ex TaqTM (DRR820A) from TaKaRa on the LightCycler 480 system provided by Roche Diagnostics in Pleasanton, California. U6 was employed as an internal reference for miRNA, while GAPDH served as an internal reference for mRNA. The amplification primers were designed and supplied by Shanghai General Biological Technology Co., Ltd., with their corresponding sequences listed in Table S1. The fold change in gene expression was calculated using the 2^−ΔΔCt^ method.

### Detection of protein expression levels by Western blot

Total proteins were extracted from rat bone tissue and osteoclasts that underwent induced differentiation. Initially, cells from each group were digested and harvested using trypsin sourced from Sigma-Aldrich (T4799-5G, Shanghai, China). Subsequently, these cells were lysed employing an enhanced RIPA lysis buffer supplemented with protease inhibitors procured from Wuhan Bodex Technology (AR0108, Wuhan, China). The protein concentration was ascertained using the BCA Protein Quantification Kit provided by Wuhan Boshido Company (AR1189, Wuhan, China). Proteins were then separated via SDS-PAGE, and the partitioned proteins were relayed onto a PVDF membrane. This membrane was then subjected to incubation at ambient temperature with 5% BSA (9048–46-8, Solarbio, Beijing, China) for an hour. Subsequent to this, the following antibodies were introduced in sequence: GFAP rabbit antibody, TRAP rabbit antibody, NFATC1 mouse antibody, MMP9 rabbit antibody, HSP70 mouse antibody, CD9 rabbit antibody, CD81 rabbit antibody, Calnexin rabbit antibody, GAPDH, and CD63. The membrane was then treated with either anti-mouse or anti-rabbit HRP secondary antibody at room temperature for another hour. ECL working solution was introduced (Omt-01, Beijing Oumijia De Medical Technology Co., Ltd., Beijing, China) for blot visualization. The gray intensity of the bands was quantified using ImageJ software, with GAPDH being utilized as the internal control.

### Hematoxylin and eosin (H&E) staining

The prepared sections are subjected to staining using the safranin staining solution (H8070, Solarbio, Beijing, China) at room temperature for 5–10 min. The slices are then rinsed with distilled water and dehydrated in 95% ethanol. Proceeding, the slices are placed into the eosin staining solution (G1100, Solarbio, Beijing, China) for 5–10 min, followed by completing the regular dehydration and permeabilization, and finally mounting the slides.

### micro-CT

The femoral bone tissue section underwent a micro-CT scan utilizing the mCT-40 system sourced from Scanco Medical, Switzerland. This examination aimed to elucidate the development of the femoral bone tissue. The operational parameters set for this scan were a current of 385μA, a voltage of 65 kV, a pixel dimension of 9 μm, a 1.0 mm AI filter, and a rotational increment of 0.4°. The image data was subsequently reconstructed using Bruker's NRecon software, and analytical procedures were carried out using CTAn software. A specific bone tissue section, measuring 0.5 mm × 0.5 mm × 0.25 mm, was isolated above the growth plate on the femoral head. The Region of Interest (ROI) was manually defined to pinpoint the targeted zone beneath the cartilage within this volume parameter. Following this, a consistent threshold range (50 ~ 255) was applied to binarize the trabecular bone, facilitating the identification of the desired region. Several micro-CT-derived parameters were evaluated, encompassing a) Bone volume fraction (BV/TV), which denotes the proportion of bone surface area relative to the tissue volume; b) Trabecular thickness (Tb. Th), an indicator of the average cross-sectional thickness reflecting structural alterations in trabecular bone; c) Trabecular number (Tb. N), representing the count of intersections between bone and non-bone tissue across a specified length; and d) BMD, which measures the concentration and distribution of mineral within the skeletal framework.

### Enzyme-Linked Immunosorbent Assay (ELISA)

The serum sample was obtained from rat venous blood through centrifugation of the coagulated blood sample at room temperature for 10 min at a centrifugal force of 2000 × g. The serum samples were subsequently stored at -80 °C. For the experiment, the CTX-I ELISA kit (YS03100B, Shanghai YaJi Biotechnology, Shanghai, China) and the TRAP5b ELISA kit (YS03672B, Shanghai YaJi Biotechnology, Shanghai, China) were utilized. The ELISA plate was read using a microplate reader (Bio-Rad) under conditions set at 450 nm, and a standard curve was constructed for subsequent data analysis.

### Isolation and identification of BMSCs

The bilateral tibia and femur of the rats were excised, and the bone marrow was subsequently flushed using modified Dulbecco's Modified Eagle Medium (DMEM)/F12 (DF-041, Sigma Aldrich, Shanghai, China) to procure a suspension of mixed cells. This mixture was centrifuged at a speed of 1000 rpm for 5 min, allowing for the isolation of the sediment-rich in BMSCs. The sedimented cells were then resuspended in DMEM/F12 and incubated at a temperature of 37 °C in a 5% CO_2_ environment. The culture medium was routinely replaced every 2–3 days. The cellular morphology was inspected utilizing transmission electron microscopy, and the Western blot method was employed to assess surface marker proteins, confirming the identity of the isolated BMSCs (Ramezani et al. 2020).

For the identification of BMSCs, a 1 × 10^6^/mL single-cell suspension was generated through a PBS wash. The suspension was then treated with fluorescently tagged antibodies, specifically CD44-FITC (MA5-17,522, Thermo Fisher Scientific), CD29-FITC (11–0291-82, Thermo Fisher Scientific), CD90-FITC (ab226, Abcam, UK), and CD45-FITC (ab33916, Abcam, UK). Following an incubation period at 4℃ for 30 min, any antibodies that did not bind were removed with a PBS wash. The samples were then analyzed using flow cytometry to determine the expression of the aforementioned labeled antibodies. In alignment with the guidelines of the BMSCs differentiation induction kit (PD-003/4/5, Procell, China), alizarin red (ARS), oil red O, and Alcian blue stainings were applied to observe the osteogenic, adipogenic, and chondrogenic differentiation potentials of the BMSCs.

### Separation and identification of osteoclasts

The rat's femur was dissected, and the epiphyses at both ends of the long bone were removed. The rinsed cells were then cultivated in the bone marrow cavity using phenol red α-MEM medium (with 10% FBS, 41,061,037, Thermofisher), supplemented with 5 ng/mL M-CSF (purchased from PeproTech, 400–28). The rinsed cells were seeded into a culture plate with 24 cell culture wells. The cells were cultured in a cell culture incubator at 37 ℃, 5% CO_2_, and saturated humidity for 3 days. Bone marrow-derived macrophages (BMMs) adhered to the cells, while the floating cells were discarded. The cells were maintained in DMEM medium (containing high glucose and 10% FBS), supplemented with 1% penicillin/streptomycin (R22148, Shanghai Yuanye Biotechnology), 20 ng/mL M-CSF, and 50 ng/mL RANKL (78,214.2, Stemcell Technologies Inc.). After 7 days, mature BMMs were evaluated using fluorescence staining with antibodies for F4/80 (ab6640, 1:200) and CD11b (ab8878, 1:50).

### Cell transfection and cell sorting

GFAP-shRNA was acquired from Thermo Fisher (USA). HEK293T cells (BFN60810479, procured from ATCC) were cultured utilizing the slow virus transfection technique in DMEM medium (Gibco) enriched with 10% FBS at 37 °C with 5% CO_2_. Both the GFAP-shRNA cell line (termed as sh-GFAP) and the control cell line (referred to as sh-NC) were established. The constructed luciferase reporter gene plasmids (sh-NC-luc, sh-GFAP-luc) were co-transfected with helper plasmids into the HEK293T cells. The target sequence specifics are provided in Table S2.

Regarding the retroviral-mediated cell transduction, BMMs were sourced from cells in the logarithmic growth phase and cultured in phenol red-free α-MEM medium enriched with M-CSF (5 ng/mL). These cells were placed in a 6-well plate at a density of 3 × 10^5^ cells per well. Upon reaching a cell confluence of 70–90%, a predetermined volume of the packaged lentivirus (with an MOI of 10 and a working titer of approximately 5 × 10^6^ TU/mL) and 5 μg/mL polybrene (TR-1003, Merck) was introduced to the culture medium to initiate transfection. Four hours post-transfection, the medium was diluted with an equivalent volume. Subsequently, 24 h post-transfection, the medium was refreshed. The transfection's efficacy was evaluated through a luciferase reporter gene 48 h post-transfection. Stably transfected cell lines were screened using a suitable concentration of puromycin (A1113803, Gibco, Grand Island, NY). After ensuring no cellular death, cells cultivated in a purine-deficient medium were gathered and subjected to an inefficiency analysis using RT-qPCR (Yan et al. 2015).

To mediate the transfection of GMNP_E_-EVs through a slow virus, logarithmic growth phase BMSC cells were seeded in a 6-well plate with 3 × 10^5^ cells per well. Once the cell confluency reached 70–90%, an appropriate amount of packaged lentivirus (MOI = 10, working titer approximately 5 × 10^6^ TU/mL) was added and transfected with the culture medium containing 5 μg/mL polybrene (TR-1003, Merck). After transfection for 4 h, the polybrene was diluted by adding an equal amount of medium. Fresh medium was replaced after 24 h of transfection. Subsequently, the transfection efficiency was assessed after 48 h using a luciferase reporter gene. EVs were captured by GMNP_E_ and cultured with BMMs using a lentivirus-mediated method.

The cells were divided into various groups: oe-NC group (lentiviral overexpression NC group), oe-GFAP group (lentiviral oe-GFAP group), sh-NC group (lentiviral silencing NC group), sh-GFAP group (lentiviral silencing GFAP group), mimic-NC-GMNP_E_-EVs + oe-NC group (GMNP_E_-EVs constructed from EVs isolated from BMSCs with lentiviral oe-NC), miR-15b-5p mimic-GMNP_E_-EVs + oe-NC group (GMNP_E_-EVs constructed from EVs isolated from BMSCs overexpressing miR-15b-5p and lentiviral oe-NC), miR-15b-5p mimic-GMNP_E_-EVs + oe-GFAP group (GMNP_E_-EVs constructed from EVs isolated from BMSCs overexpressing miR-15b-5p and lentiviral oe-GFAP), inhibitor NC-GMNP_E_-EVs + sh-NC group (GMNP_E_-EVs constructed from EVs isolated from BMSCs with lentiviral sh-NC), miR-15b-5p-inhibitor-GMNP_E_-EVs + sh-NC group (GMNP_E_-EVs constructed from EVs isolated from BMSCs with miR-15b-5p inhibitor and lentiviral sh-NC), and miR-15b-5p-inhibitor-GMNP_E_-EVs + sh-GFAP group (GMNP_E_-EVs constructed from EVs isolated from BMSCs with miR-15b-5p inhibitor and lentiviral sh-GFAP).

### TRAP staining

The TRAP staining technique was utilized to identify multinucleated osteoclasts. Once sections were deparaffinized and dehydrated, they were rinsed in distilled water for approximately 5 min. The sections were subsequently positioned inversely in a double sodium carbonate solution vessel. The container was then introduced to the anti-TRAP antibody solution, ensuring immersion for a period ranging between 5 and 20 min for thorough interaction with the sections. Excess anti-TRAP antibodies were removed, and the sections returned to their physiological conditions. Thereafter, secondary antibodies, either fluorescently labeled or enzyme-associated, were applied for detection (Chinnaiah et al. 2022; Rui et al. 2012).

For BMM cultivation, the recommended medium was α-MEM devoid of phenol red, augmented with 20 ng/mL M-CSF, 50 ng/mL RANKL, and 10% FBS. BMM culture was sustained in the medium refreshed every 3 days. The procedure for staining TRAP-positive multinucleated cells entailed the following steps: cells were initially fixed using 2.5% glutaraldehyde for 30 s before being rinsed with deionized water. After combining 0.5 mL of concentrated pomegranate red GBC solution with 0.5 mL of nitrite solution, and allowing it to stand for 2 min post-mixing, 45 ml of pre-warmed deionized water was added, followed by 0.5 mL of naphthol AS-BI phosphate solution, 2 mL of acetic acid solution, and finally, 1 mL of tartaric acid solution. Once the staining solution was prepared, it was dispersed into a 6-well plate for staining and incubated under dark conditions at 37 °C for 4 h. Post incubation, sections were counterstained with hematoxylin for 1 min, and subsequently washed with an alkaline solution until nuclei appeared a deep blue hue. Visualization and enumeration of the stained cells were conducted utilizing an inverted microscope (IX73, OLYMPUS).

### Dual-luciferase reporter gene experiment

Two plasmids, pmirGLO-GFAP-WT and pmirGLO-GFAP-MUT, were generated to incorporate the respective binding sites of miR-15b-5p and the 3'UTR of GFAP. The former represented the wild type, whereas the latter represented the mutant plasmid. HEK293T cells, procured from ATCC, were co-transfected with the miR-15b-5p mimic plasmid and the NC plasmid, NC mimic plasmid. These cells were cultured in a DMEM medium (Gibco), supplemented with 10% FBS, and incubated at 37℃ with 5% CO_2_ in a cell culture incubator. Following a 24-h transfection period, the cells were lysed and subsequently centrifuged at 12000 rpm for 1 min to collect the supernatant. Data was collected using the Dual-Luciferase Reporter Assay System (E1910, Promega), which employed a dual-luciferase reporter gene detection system. Sample activity of Firely luciferase was measured by adding 100 μL of Firely luciferase enzyme working solution, while Renilla luciferase enzyme working solution was added simultaneously to detect Renilla luciferase activity as an internal reference point.

### Separation, extraction, and identification of EVs

Upon BMSCs attaining a confluence of 80–90%, they were cultivated in serum-free MSC NutriStem® XF Media (Sartorius, Germany) for 48 h. Subsequent to this, a centrifugation process was carried out at 300 × g for 10 min, followed by another at 2000 × g for an equivalent duration to accumulate the supernatant and facilitate the elimination of deceased cells along with cellular debris. The gathered supernatant was then filtered via a 0.22 μm filter (Millipore, GVHP00010, Billerica, MA) and subjected to centrifugation at 100,000 × g for 2 h. After meticulous removal of the supernatant, the residual pellets were resuspended in 70 mL of cold PBS and centrifuged once more under the conditions of 4℃ and 100,000 × g for a 2-h duration. The PBS was then cautiously discarded. The purification process continued using the specified reagent kit and culminated with a resuspension in phosphate buffer (P1020, Solarbio, Beijing, China). This was followed by a secondary high-velocity centrifugation under identical conditions. The resultant precipitate was either stored at -80℃ for subsequent utilization or employed immediately.

Surface markers of EVs were identified employing the Western blot technique. Post the concentration process of the EVs suspension, the protein quotient was ascertained utilizing the BCA assay kit (23,227, Thermo Fisher Scientific), and an SDS-PAGE was formulated for protein denaturation and electrophoresis. Subsequently, a transfer was performed to detect the manifestation of specific EV marker proteins, namely HSP70, CD9, CD81, and Calnexin. The EV solution was incubated alongside Cy3 (S1050, Solarbio) at a ratio of 1:400 over 30 min. The DiR-tagged EVs were then acquired post centrifugation at 100,000 × g for 90 min to expel superfluous dye. This experimental procedure was reiterated thrice.

For EV identification via Transmission Electron Microscopy, 20 µL of EVs were deposited on a copper mesh and allowed to rest for 3 min, after which the surplus liquid was siphoned using filter paper. Subsequent to this, 30 μL of phosphotungstic acid solution (with a pH of 6.8) was added, and a room-temperature incubation ensued for 5 min. After drying under an illuminating lamp, visuals were captured employing a transmission electron microscope (Hitachi H-7650, sourced from Shanghai Bahe Instrument Technology Co., Ltd.). The dimensional distribution of the EVs was gauged utilizing a nanotechnology particle analysis instrument, specifically the Zeta View_Particle Metrix, acquired from Dacang Huajia Scientific Instruments.

### Synthesis and characterization of GMNPs

To generate GMNPs or GMNPs labeled with RhB, the progressive immobilization of different chemical substances on the surface of Fe_3_O_4_@SiO_2_ core–shell magnetic nanoparticles (MNPs) was conducted. Initially, MNPs were dispersed in 100 mL anhydrous ethanol, followed by the addition of 2.5 mL of either MPTS solution (50 wt% ethanol) or a mixed solution of 2,3-dimethyl maleic anhydride/MPTS (molar ratio 1:9) and RhB in ethanol (50 wt%) to the suspension. The mixture was stirred for 8 h and subsequently filtered. The resulting solid was washed with ethanol and dried under vacuum, resulting in the production of Fe_3_O_4_@SiO_2_-C = C with or without an RhB label.

Next, Fe3O4@SiO_2_-C = C was dissolved in a mixture of methanol and tetrahydrofuran (THF) (1:1 v/v) in a 20 mL flask. Then, 1 mL of mercaptopropionic acid was added and stirred for 10 min. Afterward, 60 mg of 2,3-dimethyl maleic anhydride was added, and the solution was irradiated under UV light (365 nm) for 1 h while being stirred to initiate the thiol-ene reaction. Following this, the lights were turned off, and stirring of the solution was continued overnight. The resulting product was collected by centrifugation, washed multiple times with ethanol, and dried under vacuum at 60 °C for 12 h, resulting in the production of Fe_3_O_4_@SiO_2_-NH-NH_2_ with or without RhB labeling.

Subsequently, Fe_3_O_4_@SiO_2_NH-NH_2_ nanoparticles (50 mg) were mixed thoroughly with methanol (20 mL) containing CHO-PEG 4000-CHO (1 g) and stirred at room temperature for 12 h. After adding 20 μL of ice-cold acetic acid, the mixture was further stirred for 12 h. After washing the modified nanoparticles with ethanol and water, the particles were dried under vacuum at 60 °C for 12 h, leading to the production of Fe_3_O_4_@SiO_2_-PEG-CHO (GMNPs or GMNPs labeled with RhB).

The intermediate and final products were analyzed using scanning electron microscopy (SEM) (S-4800, Hitachi, acquired from Shanghai Fulei Optical Technology Co., Ltd.), energy-dispersive spectroscopy (IQLAAHGABMFAAWMACL, Thermo Fisher), and Fourier-transform infrared spectroscopy (912A0770, Thermo Fisher). The magnetization curve was recorded using the Micromag model 2900 alternating gradient magnetometer (Princeton Measurements Cooperation). X-ray diffraction patterns were obtained using copper Kα irradiation in the RINT2000 vertical wind velocity meter (Rigaku). The size and size distribution of particles were measured using dynamic light scattering (DLS) (Nano ZS90, Malvern).

### Preparation and biocompatibility study of GMNPE

The GMNP_E_ was formulated by amalgamating the anti-CD63 antibody (PA5-92,370, Thermo Fisher) with aldehyde-functionalized MNPs, designated as Fe_3_O_4_@SiO_2_PEG-CHO or simply GMNP. Initially, the GMNP solution was purified via magnetic separation, ensuring a concentration of 1 mg per milliliter, and subsequently resuspended in PBS. The anti-CD63 antibody was introduced and a thorough amalgamation was ensured. This compound was then incubated at 4 °C with rotational agitation overnight to yield GMNP_E_. For the fabrication of a fluorescently labeled GMNP_E_, a similar procedure was adhered to, but GMNPs marked with RhB were utilized in lieu of the conventional GMNPs.

After the antibody attachment, the GMNP_E_ was thrice rinsed using PBS and preserved at a temperature of 4 °C. A comparative analysis of MNPs, GMNPs, and GMNPE was conducted via TGA to assess the antibody's loading capacity onto GMNP_E_ via TGA (utilizing STA 6000, Perkin Elmer). Both MNP_E_ and GMNP_E_ were co-incubated with HSA (sourced as MABX1932 from Sigma Aldrich, Shanghai, China) for three days at 37 °C. The adsorptive capacity of HSA was ascertained using FITC-tagged HSA through fluorescence spectroscopy. Additionally, the stability of HSA was scrutinized. The adsorption capacity percentage was computed by dividing the quantity of HSA that had adhered to the nanoparticles by the overall quantity of HSA that was adsorbed.

### Preparation and characterization of GMNPE-EVs

The fluorescent labeling of purified EVs was conducted using the green PKH67 membrane dye (MINI67, purchased from Shanghai Sigma Aldrich Trading Co., Ltd). To collect EVs, the GMNP_E_ nanoparticles mentioned above were incubated with suspended EVs or serum at 37 °C. After a 2-h incubation, magnetic separation was performed to obtain GMNP_E_-EVs, which were then subjected to three washes with PBS. The morphology of the resulting GMNP_E_-EVs was observed using a high-resolution transmission electron microscope (200 kV, CM200, Philips). Both GMNP_E_ and EVs were fluorescently labeled with RhB and PKH67 to confirm their binding relationship.

To assess the binding ability of GMNP_E_ to EVs, the reduction of EVs in the serum before and after co-culture was calculated. The obtained data was compared with the corresponding GMNP_N_ values of the IgG control dose to determine the relative collection amount of EVs. The total protein concentration in EVs was quantified using the BCA protein assay method (71,285-M, purchased from Shanghai Sigma Aldrich Trading Co., Ltd.). The percentage of vesicle binding capacity was calculated by dividing the number of vesicles by the number of nanoparticles used for capture.

The magnetic separation of EVs was confirmed through Western blot analysis. Briefly, positive control EVs, GMNP_E_-EVs, and GMNP_N_-EVs were prepared using Radioimmunoprecipitation Assay (RIPA) buffer (20–188, purchased from Sigma-Aldrich Trading Co., Ltd. Shanghai). The solution was removed through centrifugation, and separation was carried out using SDS-PAGE, which was subsequently transferred to PVDF membranes. The membrane was washed and blocked at 4 °C for 1 h, followed by incubation overnight with the primary antibody CD63 (rabbit anti, PA5-92,370, Thermo Fisher) at room temperature. Subsequently, the membrane was washed with TBST for 5 min, repeated three times, and then incubated and developed with the goat anti-rabbit IgG secondary antibody (ab205718, 1:20,000 dilution, Abcam) and the developing solution. Finally, the protein analysis was performed using ImageJ 1.48 software.

### Phagocytosis of GMNP_E_-EVs by osteoclasts

The uptake proficiency of osteoclasts for GMNP_E_-EVs was assessed by co-culturing RhB-marked GMNP_E_ with PKH67-tagged EVs at 37 °C alongside osteoclasts for three days. Following the incubation, the culture was rinsed three times using PBS and subsequently fixed using polyformaldehyde for 15 min. To ascertain the intracellular localization of GMNP_E_-EVs, the cytoskeleton and cell nuclei were stained with Hoechst 33,342, procured from Sigma Aldrich, Shanghai, China.

### Immunofluorescent staining

GMNP_N_ and GMNP_E_ were immobilized in 4% PFA solution at room temperature and underwent a 30-min treatment for permeabilization and blocking. They were then incubated with anti-CD63 antibody (PA5-92,370, Thermo Fisher) at a dilution of 1:100 at 4 °C. On the following day, the cells were washed three times with a Blank solution and subsequently incubated with Goat Anti-Rabbit Antibody coupled with Alexa Fluor® 555 (#60,839, 1:50 dilution, CST) without light exposure. Finally, the cells were observed under a fluorescent microscope (Zhao et al. 2018; Guan et al. 2020).

RhB-labeled GMNP_E_-EVs and PKH67-labeled EVs were injected into the bodies of DO rats. N52 neodymium magnets were then employed to apply an external magnetic field at the femur site in the rats, facilitating the accumulation of flowing GMNP_E_ nanoparticles at the femur site. After the rats were euthanized, their femurs, brains, livers, kidneys, and other tissues were extracted and prepared for longitudinal cryosectioning. A fluorescence microscope (80i, purchased from Shanghai Henghao Instruments Co., Ltd.) was utilized to observe these tissues.

### CCK-8 assay

During their logarithmic growth phase, osteoclasts were plated in a 96-well plate at a concentration of 5 × 103 cells per well. Subsequently, 10 μL of the CCK-8 reagent solution (C0038, Beyotime Biotechnology Co., Ltd., Shanghai, China) was introduced to each well. The plate was then incubated in a humidified chamber set at 37℃. An hour post-incubation, the absorbance of each well was measured at 450 nm using a Micro-plate Reader (abx700005, Beijing Qiwei Yicheng Technology Co., Ltd.), and the results were meticulously recorded.

### Statistical analysis

GraphPad Prism 8.0 software was utilized to process all the data. The data is presented as the mean ± standard deviation (mean ± SD). An independent t-test was employed to compare the two groups, whereas a one-way analysis of variance (ANOVA) was used to compare data among multiple groups. Levene's test was conducted to assess the homogeneity of variances. If the variances were found to be homogeneous, pairwise comparisons were performed using Dunnett's t-test and LSD-t-test. In cases where the variances were unequal, Dunnett's T3 test was employed. A p-value below 0.05 indicated statistical significance in the comparisons between the two groups.

## Results

### Identification of critical genes in DO using the GEO database

**Fig. 1:**
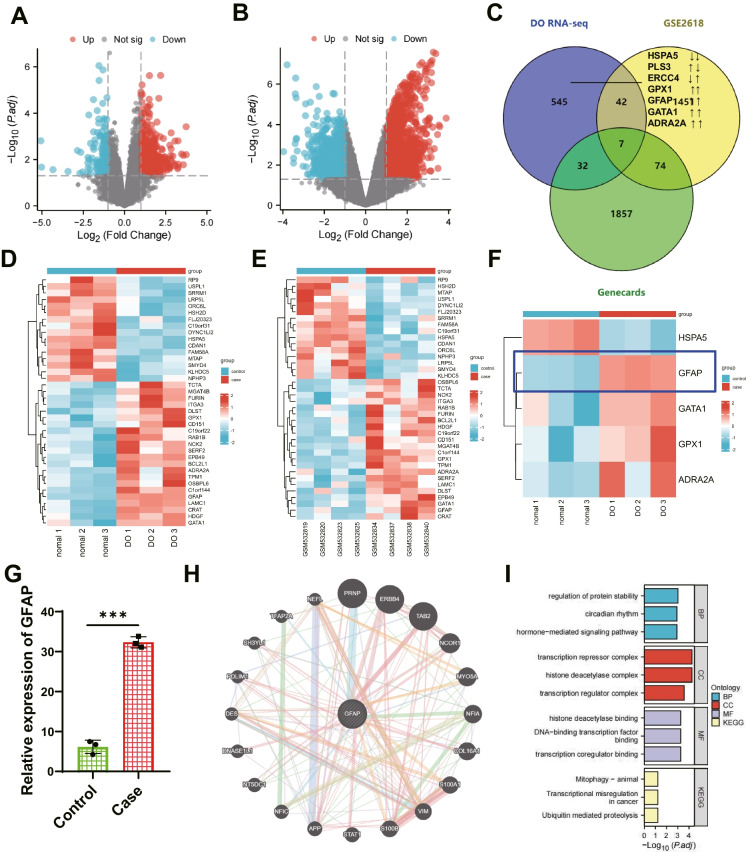
Selection of essential genes related to DO in the database. Note: (**A**) Volcano plot of DEGs analysis based on RNA-seq data set (Control group: n = 3; Case group: n = 3). Red dots represent up-regulated genes, blue dots represent downregulated genes, and gray dots represent genes with no difference; (**B**) Volcano plot of DEGs analysis based on GSE26168 data set (Control group: n = 4; Case group: n = 4); (**C**) Venn diagram showing the intersection of DEGs in the GSE56116 and GSE35958 microarray data sets with diabetes-related genes; (**D**) Heatmap of the 38 intersection genes in the RNA-seq data set (Control group: n = 3; Case group: n = 3); (**E**) Heatmap of the 38 intersection genes in the GSE26168 data set (Control group: n = 4; Case group: n = 4); (**F**) Differential expression of 5 intersection genes in RNA-seq data (Control group: n = 3; Case group: n = 3); (**G**) Bar chart of differential expression of GFAP in the RNA-seq data set; (**H**) Protein–protein interaction (PPI) network diagram of GFAP interactions; (**I**) KEGG functional enrichment analysis, including molecular function (MF), biological process (BP), and cell component (CC)

We constructed a DO rat model to identify essential genes regulating DO and obtained peripheral blood RNA-seq dataset through high-throughput transcriptome sequencing. GEO database retrieval obtained the transcriptome RNA sequencing data chips GSE26168 related to diabetes. The DO-related RNA-seq dataset includes RNA expression profiles of peripheral blood from 3 regular and 3 DO rats, totaling 626 DEGs. Four hundred twenty-nine were up-regulated, and 197 were down-regulated (Fig. 1A). GSE26168 includes RNA expression profiles of peripheral blood from 4 healthy subjects and 4 diabetic patients, with 1574 DEGs. Nine hundred seventy-six were up-regulated, while 598 were downregulated (Fig. 1B). After taking the intersection of DEGs from two chips, 49 intersected genes were obtained (Fig. 1C), with only 22 genes consistently up-regulated, and 16 genes consistently downregulated. Differential heat map analysis (Fig. 1D-E) was performed for 38 genes, showing consistent trends using two microarray chips.

At the same time, DO-related genes were searched on the GeneCards website, with a Relevance score greater than 5 as the filtering criteria. In the end, a total of 1970 DO-related genes were obtained. The intersection of DEGs from two datasets and DO-related genes resulted in 7 overlapping genes (Fig. 1C). HSPA5 was consistently down-regulated, while GPX1, GFAP, GATA1, and ADRA2A were up-regulated. However, PLS3 and ERCC4 showed inconsistent expression trends in both datasets. Literature has reported that the GPX1 gene can mediate mitochondrial antioxidant function and promote the repair of osteoporotic bone defects (Zhou et al. 2023). Many studies have shown the relationship between GFAP and neurological disorders such as gliomas (Middeldorp and Hol 2011). GFAP is associated with diabetes (Timper et al. 2020). However, detailed reports on the association between osteoporosis and the GATA1 gene with bone marrow fibrosis have yet to be found (Gilles et al. 2017). ADRA2A is closely related to diabetes and is involved in the neuroendocrine regulation of bone resorption (Mlakar et al. 2015). GFAP expression was found to be the most among the five genes, showing consistent trends in the DO-related RNA-seq dataset (Fig. 1F), and GFAP exhibited higher expression in the DO-related RNA-seq dataset (Fig. 1G). Therefore, this study aims to investigate the mechanism of GFAP in DO by focusing on its target gene.

To explore the interaction of GFAP with other proteins, we used the String website to generate the GFAP protein–protein interaction network. Then, we visualized the network using Cytoscape software (Fig. 1H), resulting in 21 interacting proteins. After conducting GO enrichment analysis, it was found that the main enrichments in BP were regulation of protein stability, circadian rhythm, hormone-mediated signaling pathway, and other terms. The main entries in CC are the i-transcription repressor complex, histone deacetylase complex, transcription regulator complex, and so on. Enrichment in MF is mainly observed in entries such as histone deacetylase binding, DNA-binding transcription factor binding, and transcription coregulator binding. Enrichment in KEGG is mainly observed in entries such as Mitophagy—animal, Transcriptional misregulation in cancer, and Ubiquitin mediated proteolysis (Fig. 1I). In conclusion, GFAP is a critical gene in DO and exhibits high expression in DO.

### GFAP promotes osteoclastogenesis in DO rats

Recent studies have found that there is also an expression of GFAP in osteocytes, which is closely associated with bone metabolism processes (Li et al. 2020). Current research indicates that GFAP may be involved in osteoclast differentiation and function by regulating intracellular kinase signaling pathways (Glavan et al. 2021). Meanwhile, the expression of GFAP is also associated with the occurrence and progression of diseases such as osteoporosis.

**Fig. 2:**
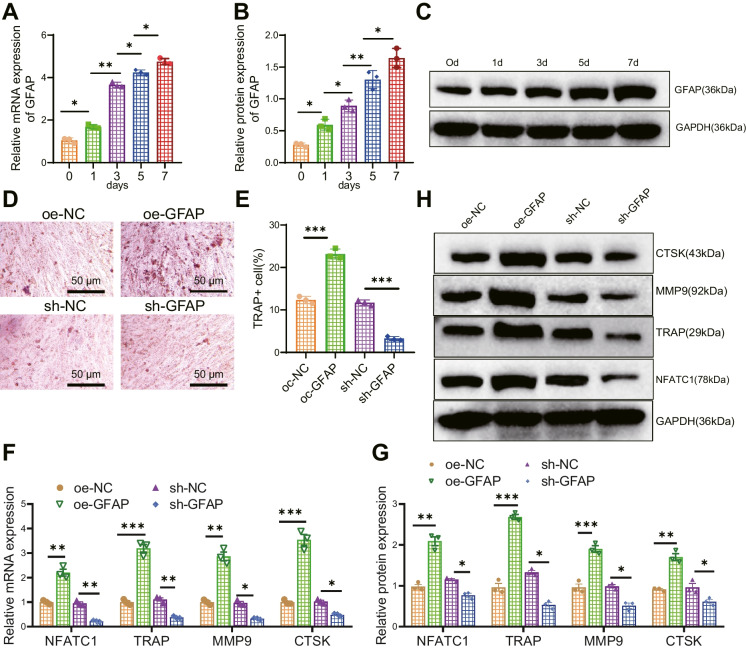
Influence of GFAP on osteoclast differentiation. Note: (**A**-**C**) RT-qPCR and Western blot analysis of GFAP expression levels at different time points in BMMs stimulated with RANKL; (**D**-**E**) TRAP staining to detect osteoclast differentiation in different groups of BMMs (MNC ≥ 3 nuclei were considered positive), scale bar = 100 μm; (**F**-**G**) RT-qPCR and Western blot analysis of TRAP, NFATC1, MMP9, and CTSK expression levels in different groups of BMMs. * indicates *P* < 0.05 compared to the control group, ** indicates *P* < 0.01, *** indicates *P* < 0.001. The cell experiments were repeated three times

To investigate the relationship between GFAP and osteoclast formation, firstly, we isolated BMMs from rat bone marrow and confirmed that nearly all cells express macrophage-specific surface markers CD11b and F4/80 (Figure S1A-B), indicating successful isolation of BMMs. Subsequently, we detected the expression levels of GFAP in BMMs stimulated with M-CSF and RANKL for 1, 3, 5, and 7 days using RT-qPCR. We found that during the osteoclast formation induced by RANKL, both GFAP mRNA and protein levels increased and showed a time-dependent pattern (Fig. 2A-C). Overexpression or knockdown of GFAP in BMMs was achieved using lentivirus. Subsequently, the cells were co-cultured with M-CSF and RANKL for 7 days. The expression levels of GFAP were detected using RT-qPCR, revealing a higher silencing efficiency in GFAP-sh-1 compared to GFAP-sh-2. Therefore, GFAP-sh-1 was selected for further experiments (Figure S2A-B). The TRAP staining results revealed that the overexpression of GFAP increased the number of TRAP-positive multinucleated cells (MNCs) (≥ 3 nuclei), while silencing GFAP reduced the number of TRAP-positive cells (Fig. 2D-E). In addition, the results of RT-qPCR and Western blot detection showed that the expression levels of bone resorption marker genes TRAP, NFATC1, MMP9, and CTSK, as well as proteins, were increased in BMMs overexpressing GFAP while silencing GFAP showed the opposite trend (Fig. 2F-G).

To further investigate the relationship between GFAP and bone formation in DO rats, we created a DO rat model and used H&E staining to observe the conditions of the femoral diaphysis in normal rats and DO rats. The staining results showed that the trabecular bones at the diaphysis of normal rats were thick, well-arranged, and relatively dense, with clear and intact structures. In contrast, the diaphysis of DO rats' femurs exhibited the opposite characteristics (Figure S3A). The number of trabeculae (Tb. N), trabecular thickness (Tb. Th), bone volume/total volume (BV/TV), and BMD in the distal femur of rats were observed using Micro-CT. The results showed that the DO rats had thinner trabecular bone and disorganized trabecular structure, decreased interconnectivity, and increased separation, and thinning of cortical bone compared to the control rats.

Additionally, BV/TV, Tb. N, Tb. Th, and BMD were all reduced, indicating successful modeling of DO rats (Figure S3B-C).

**Fig. 3:**
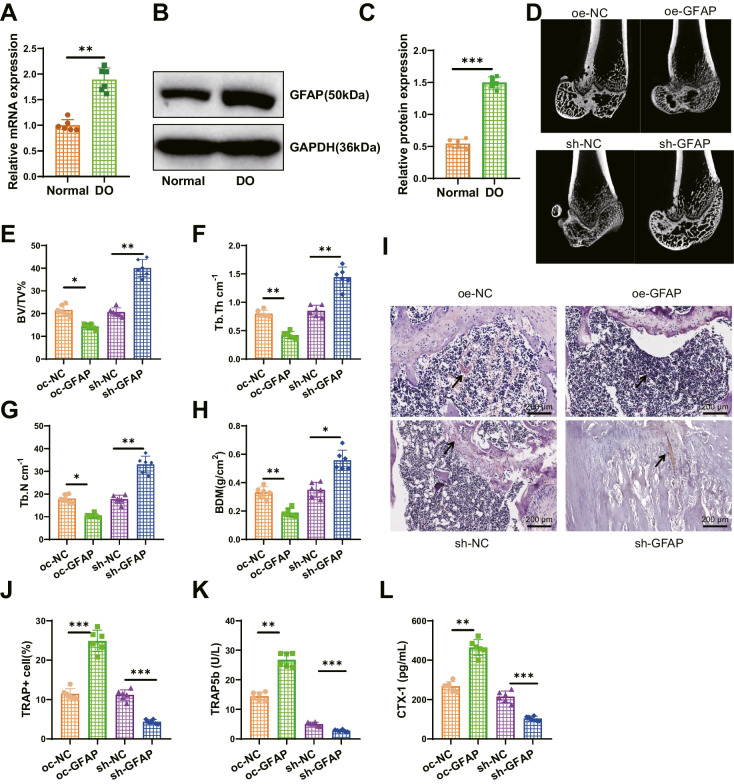
Influence of GFAP on osteoporosis in DO rats. Note: (**A**-**B**) RT-qPCR (**A**) and Western blot (**B**-**C**) analysis of GFAP expression levels in bone tissues of different groups of rats; (**D**) Micro-CT analysis of femurs in different groups of rats; (**E**–**H**) Statistical analysis of BV/TV, Tb. N, Tb.Th, and BMD in femurs of different groups as shown in panel D; (**I**) TRAP staining to detect the number of osteoclasts in femoral tissues of different groups, with arrows pointing to positive cells, scale bar = 200 μm; (**J**) Statistical analysis of the number of positive osteoclast cells shown in panel I; (**K**-**L**) ELISA to measure the levels of bone resorption markers TRAP5b (**K**) and CTX-I (**L**) in serum of different groups of rats. Each group consisted of 6 rats. * indicates *P* < 0.05 compared to the control group, ** indicates *P* < 0.01, *** indicates *P* < 0.001

The expression level of GFAP in the bone tissue of DO rats was detected using RT-qPCR and Western blot techniques. The results showed increased GFAP expression in DO rats compared to normal rats (Fig. 3A-C). Next, in rats, overexpressing or silencing GFAP intracellularly, RT-qPCR detection results showed that overexpression of GFAP increased the expression levels of GFAP while silencing GFAP decreased the expression levels of GFAP (Figure S3D-E). The results of Micro-CT observation showed that overexpression of GFAP further aggravated osteoporosis in DO rats while silencing GFAP alleviated osteoporosis in DO rats (Fig. 3D-H). Subsequently, TRAP staining was utilized to detect the number of osteoclasts in the femoral tissue. The results revealed that overexpression of GFAP increased the number of TRAP-positive cells while silencing of GFAP reduced the number of TRAP-positive cells (Fig. 3I-J). Meanwhile, an ELISA assay was performed to detect serum bone resorption marker CTX-I and osteoclast marker TRAP5b levels. The results showed that overexpression of GFAP increased serum CTX-I and TRAP5b; in contrast, silencing GFAP reduced serum CTX-I and TRAP5b (Fig. 3K-L).

The above results indicate that GFAP can promote osteogenesis in DO rats.

### Delivery of EVs can inhibit the expression of GFAP in osteoclasts by transferring miR-15b-5p

**Fig. 4:**
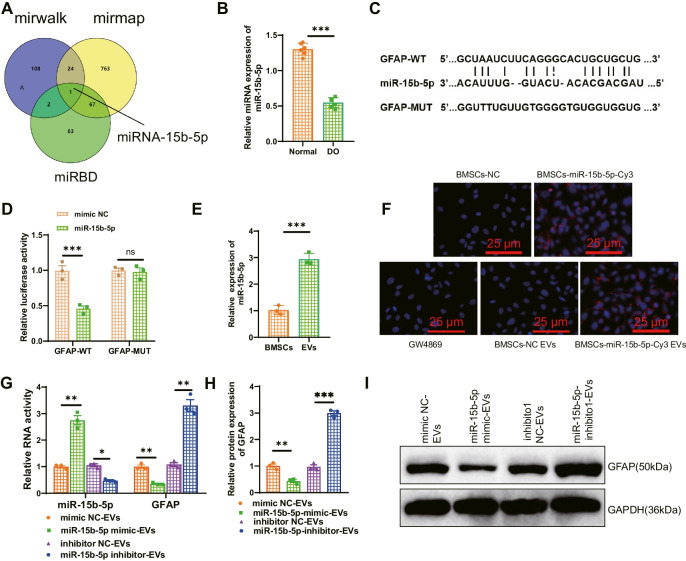
EVs-mediated regulation of GFAP expression by miR-15b-5p. Note: (**A**) Prediction of miRNA associated with GFAP using the Norwalk, minimap, and miRDB databases, with the middle section representing the intersection of the three data sets; (**B**) RT-qPCR analysis of miR-15b-5p expression levels in bone tissues of different groups of rats; (**C**-**D**) Dual-luciferase reporter gene experiment to verify the targeted binding relationship between miR-15b-5p and GFAP; (**E**) RT-qPCR analysis of miR-15b-5p expression in BMSCs and EVs; (**F**) Fluorescence microscopy to detect Cy3-labeled fluorescence signals (Scale bar = 20 μm); (**G**) RT-qPCR analysis of miR-15b-5p and GFAP expression levels in osteoclasts; (**H**-**I**) Western blot analysis of GFAP expression levels in different groups of osteoclasts. Each group consisted of 6 rats. The cell experiments were repeated three times. * indicates *P* < 0.05 compared to the control group, ** indicates *P* < 0.01, *** indicates *P* < 0.001

To better understand the biological role of GFAP in DO, we first used the MirWalk, miRmap, and miRBD databases to obtain upstream miRNAs of GFAP and conducted a Venn analysis. After screening, a common miRNA, namely miR-15b-5p (Fig. 4A), was obtained. GFAP, hsa-miR-15b-5p, and rno-miR-15b-5p all have binding sites, and miR-15b-5p is downregulated in diabetes (Xu et al. 2019). Therefore, we speculate that miR-15b-5p may suppress the expression of GFAP.

To validate the above conjecture, we first examined the expression level of miR-15b-5p in the bone tissue of DO rats. The results of RT-qPCR detection showed that, compared with the Normal group, the expression level of miR-15b-5p in the DO group decreased (Fig. 4B). We also conducted a dual-luciferase reporter assay to detect the binding between rno-miR-15b-5p and GFAP. The results showed that compared with the mimic NC group, the luciferase activity of the GFAP-WT co-transfection group was decreased in the miR-15b-5p mimic group, while there was no difference in the luciferase activity of the GFAP-Mut co-transfection group in the miR-15b-5p mimic group (Fig. 4C-D). It indicates that miR-15b-5p can target and inhibit the expression of GFAP.

EVs can act as natural nanocarriers to deliver biological information such as miRNA and lncRNA (Song et al. 2019). Therefore, we aimed to use EVs to carry miR-15b-5p for further studies. First, we induced adipogenic, osteogenic, and chondrogenic differentiation of BMSCs (Figure S4A-B) and detected positive expression of CD90, CD29, and CD44 in BMSCs, and negative expression of CD45 (Figure S4C). Subsequently, we obtained EVs derived from BMSCs through high-speed centrifugation and identified their morphology using TEM. The results show that BMSCs-EVs are membrane-bound vesicles with a similar circular or elliptical shape (Figure S4D), and NTA analysis indicates an average diameter of approximately 120 nm for the EVs (Figure S4E). Western blot was used to detect specific surface markers CD9, HSP70, and CD81 expression in EVs. The results showed high expression of these markers, while Calnexin was not expressed (Figure S4F), indicating successful isolation of EVs. RT-qPCR analysis showed that the level of miR-15b-5p in EVs derived from BMSCs was higher than that in BMSCs (Fig. 4E), indicating an enrichment of miR-15b-5p in EVs.

To further demonstrate that EVs can deliver miR-15b-5p into cells, we co-cultured BMSCs transiently transfected with Cy3-labeled miR-15b-5p mimic with osteoclasts. After 3 days of indirect co-culture, osteoclasts exhibited Cy3 fluorescence. We then treated BMSCs with EV release inhibitor GW4869 and found that the fluorescence of Cy3 in osteoclasts disappeared. However, the fluorescence signal remained in osteoclasts incubated with EVs isolated from BMSCs transfected with miR-15b-5p mimic (Fig. 4F). The above results indicate that miR-15b-5p can shuttle between cells and enter osteoclasts through EVs secreted by BMSCs.

Subsequently, we detected the expression of miR-15b-5p and GFAP through RT-qPCR and obtained the following results: Compared with the mimic NC-EVs group, the miR-15b-5p mimic-EVs group showed upregulation of miR-15b-5p and downregulation of GFAP in osteoclast cells; Compared with the inhibitor NC-EVs group, the miR-15b-5p inhibitor-EVs group exhibited low expression of miR-15b-5p and upregulation of GFAP in osteoclast cells. Western blot analysis showed that compared with the mimic NC-EVs group, the miR-15b-5p mimic-EVs group decreased the expression of GFAP protein. Compared with the inhibitor NC-EVs group, the miR-15b-5p inhibitor-EVs group increased the expression of GFAP protein (Fig. 4G-I). The above results indicate that EVs can deliver miR-15b-5p to osteoclasts and further inhibit the expression of GFAP.

### Synthesis and characterization of GMNPE

Due to the enrichment of miRNA-mediated cell communication, EVs are essential in maintaining tissue homeostasis and pathological processes. Functional implementation relies heavily on the biological distribution of EVs within the organism, which is limited by tissue/cell-specific delivery. MNPs have been widely used for precise targeting and molecular delivery, so we speculate whether it is possible to manipulate the biological distribution of EVs using MNPs to achieve therapeutic effects (Liu et al. 2020).

**Fig. 5:**
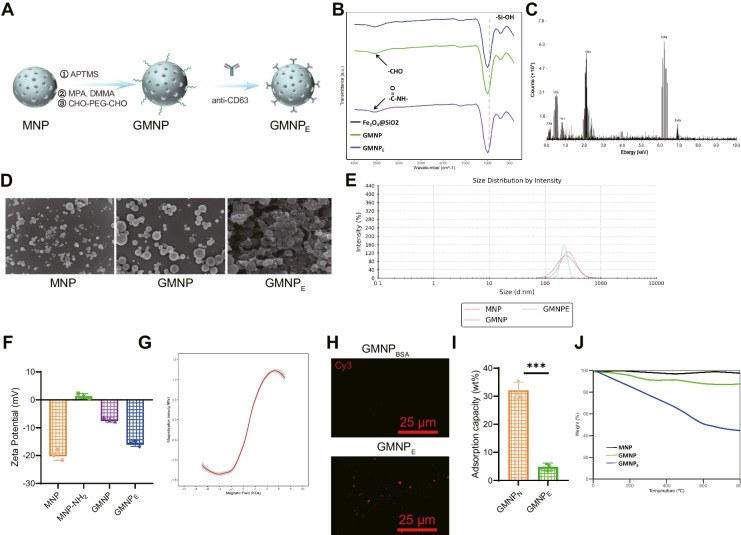
Characterization of GMNP_E_-EVs. Note: (**A**) Schematic diagram of GMNP_E_-EVs formation; (**B**) Infrared spectroscopy analysis of the chemical composition at each step of GMNP_E_ formation; (**C**) Elemental analysis by energy-dispersive X-ray spectroscopy (EDS); (**D**) SEM observation of MNP, GMNP, and GMNP_E_ (scale bars = 1 μm); (**E**) Particle size distribution of GMNP_E_ at key steps of formation; (**F**) Changes in Zeta potential of GMNP_E_ before and after stepwise modification; (**G**) Magnetic hysteresis loop of GMNP_E_; (**H**) Confocal microscopy imaging of anti-CD63 on GMNP_E_ surface (bar = 25 μm); (**I**) Measurement of human serum albumin (HSA) adsorption on GMNP_E_ and GMNP_N_ nanoparticles after incubation at 37 °C for 3 days, FITC-labeled HSA, fluorescence spectroscopy used to measure the adsorption amount of HSA; (**J**) TGA analysis of antibody content in GMNP_E_. All experiments were repeated three times. * indicates difference between two groups with *p* < 0.05, ** indicates difference with *p* < 0.01, *** indicates difference with *p* < 0.001

Extensive research has shown that iron oxide nanoparticles can control drug delivery through external magnetic fields to treat various diseases, including osteoporosis (Tran and Webster 2013; Schneider and Husz 1986). As a result, we synthesized nanoparticles composed of a magnetically guided iron oxide () core, a silicon dioxide (SiO_2_) shell, and a stimuli-responsive poly(ethylene glycol) (PEG) corona (Fig. 5A). The infrared spectrum of GMNP_E_'s chemical composition reveals the expected characteristic peaks of functional groups in each synthetic step, including modifying Fe_3_O_4_ nanoparticles with -Si–O-H, -NH_2_, and aldimine condensation (Fig. 5B). We also performed energy-dispersive X-ray spectroscopy on GMNPs to evaluate their elemental composition. The apparent signals of Fe, Si, C, and N indicate the presence of Fe_3_O_4_, SiO_2_, PEG, and hydrazine bonds in the GMNPs (Fig. 5C). SEM images show that the size of spherical Fe_3_O_4_@SiO_2_ nanoparticles (MNPs) increases after polymer (PEG) modification at 200 nm, and the surface roughness changes from rough to relatively smooth (Fig. 5D). The particle size further increases after binding with anti-CD63, confirmed by particle size analysis (Fig. 5E) in line with SEM results. Then, zeta potential measurement was used to investigate the stepwise modification of GMNP_E_. The results showed that the surface charge gradually changed based on the electron properties of different functional groups (Fig. 5F). The magnetic measurements of GMNP_E_ particles indicate that the nanospheres exhibit superparamagnetic behavior and can achieve magnetic enrichment (Fig. 5G).

Next, we identified the antigens on the GMNP_E_ surface using anti-rat Cy3-Red secondary antibodies. Compared to GMNPBSA, the presence of CD63 was confirmed in GMNP_E_ (Fig. 5H). Next, we cultured GMNP_N_, GMNP_E_, and HSA at 37 °C for 3 days and determined the adsorption of HSA using FITC-labeled HSA through fluorescence spectroscopy. The adsorption of HSA on GMNP_E_ was reduced compared to GMNP_N_, primarily due to the majority of aldehyde groups on the surface of GMNP_E_ binding with anti-CD63 (Fig. 5I). Thermogravimetric analysis (TGA) was used to evaluate the antibody loading capacity of GMNP_E_. The mass percentage of anti-CD63 bound to GMNPs was determined as 34% based on the difference in final residual weights between GMNPs and GMNP_E_ (Fig. 5J). The above results demonstrate the successful synthesis of MNP-based materials, GMNP_E_, that can carry antibodies.

### GMNPE-carrying EVs enrich the bone tissue of DO rats

**Fig. 6:**
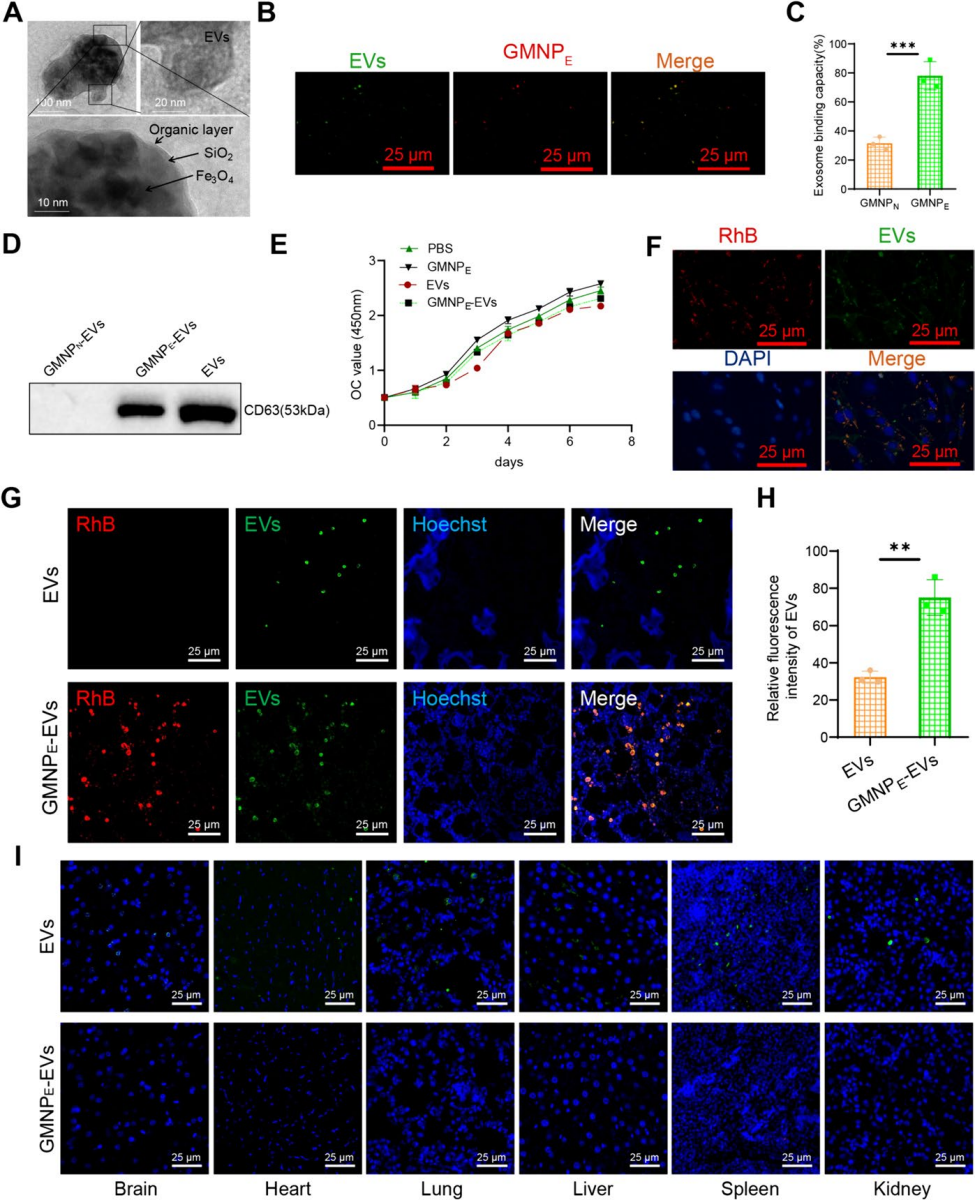
Enrichment of GMNP_E_-EVs in DO rat bone tissue. Note: (**A**) TEM imaging showing the clear core–shell corona structure of GMNP_E_ nanoparticles and the double-layer membrane structure of EVs, top left bar = 100 nm, top right bar = 20 nm, bottom right bar = 10 nm; (**B**) Representative confocal microscopy imaging of GMNP_E_-EVs, co-localization of MNPs (red) and EVs (green), bar = 25 μm; (**C**) Binding capacity of GMNP_E_ and GMNP_N_ with EVs; (**D**) Western blot analysis of CD63 expression in GMNP_N_-EVs, GMNP_E_-EVs, and EVs lysates; (**E**) CCK-8 assay to evaluate the effect of nanoparticles on osteoclast proliferation; (**F**) Confocal microscopy images showing the localization of GMNP_E_-EVs in cells, bar = 25 μm; (**G**) Confocal microscopy images showing the localization of GMNP_N_-EVs and GMNP_E_-EVs in rat body, bar = 25 μm; (**H**) Relative fluorescence intensity of EVs in image G; (**I**) Immunofluorescence detection of EVs (green) and GMNP_E_ (red)-EVs (green) signals in brain, heart, lung, liver, spleen, and kidney tissue cells, bar = 25 μm. Each group consisted of 3 rats, and cell experiments were repeated 3 times. * indicates difference between two groups, ** indicates difference with *p* < 0.01, *** indicates difference with *p* < 0.001

To identify whether GMNP_E_ can specifically load EVs and mediate targeted intercellular communication. TEM imaging analysis demonstrated that GMNP_E_-EVs incubated with EVs derived from rat BMSCs exhibited a clear nuclear envelope-like structure and evident vesicular structures (Fig. 6A). Further confirmation of GMNP_E_ (labeled with RhB) capturing EVs (labeled with PKH67) was achieved through fluorescence confocal microscopy, where co-localization of EVs-associated fluorescence (green) and GMNP_E_-associated fluorescence (red) was observed (Fig. 6B).

GMNP combined with IgG antibodies as a control (GMNP_N_) was further used to quantify the binding ability of EVs to GMNP_E_. The results showed an enhancement in EVs captured by GMNP_E_ compared to GMNP_N_ (Fig. 6C). Similarly, GMNP_N_, GMNP_E_, and EVs were co-cultured, and then lysates of GMNP_N_-EVs, GMNP_E_-EVs, and EVs (as a positive control) were obtained separately. The expression of CD63 was detected using the Western blot technique. The results showed that CD63 could be detected in both GMNP_E_-EVs and EVs but not in GMNP_N_-EVs (Fig. 6D).

To assess the toxic effects of nanoparticles on osteoclasts, we co-cultured GMNP_E_, EVs, and GMNP_E_-EVs with logarithmic-phase osteoclasts separately. CCK-8 cell proliferation assay showed that GMNP_E_ had minimal effects on cell proliferation compared to the PBS control group. However, compared to the GMNP_E_ group, GMNP_E_-EVs and EVs exhibited a slight decrease in cell proliferation but with no difference. It suggests that EVs may have a specific inhibitory effect on osteoclast proliferation (Fig. 6E). Subsequently, further observation was conducted to determine whether osteoclasts could absorb the nanoparticles. We co-cultured GMNP_E_-EVs (GMNP_E_ labeled with RhB fluorescence (red) and EVs labeled with PKH67 fluorescence (green)) with osteoclasts for 3 days. Confocal microscopy images revealed that GMNP_E_ could transport EVs and enter osteoclasts (Fig. 6F).

Next, we intravenously injected EVs and GMNP_E_-EVs (RhB-labeled GMNP_E_ in red and PKH67-labeled EVs in green) into DO rats through the tail vein. Using N52 neodymium magnets, an external magnetic field is applied to the rats' femur site, causing the accumulation of GMNP_E_ nanoparticles circulating in the blood at the femur site. Confocal microscopy images show that a small number of EVs can be observed in the trabecular bone of DO rats in the EVs injection group. In the GMNP_E_-EVs injection group, both GMNP_E_ and EVs can be simultaneously observed in the trabecular bone (Fig. 6G), and the fluorescence intensity reveals that a higher concentration of EVs is enriched in the trabecular bone of DO rats in the GMNP_E_-EVs injection group (Fig. 6H). EVs injected into other tissues of SD rats, such as the brain, heart, lungs, liver, spleen, and kidneys, were observed in small amounts. However, almost no GMNP_E_-EVs signal was detected in other tissues of SD rats injected with GMNP_E_-EVs, demonstrating the targeted delivery of GMNP_E_-EVs (Fig. 6I).

The above results indicate that GMNP_E_ can successfully transport EVs to enrich the bone tissues of DO rats.

miR-15b-5p-GMNP_E_-EVs can inhibit osteoclast formation by targeting GFA.

We have validated in the above experiment that rat osteoclasts can uptake GMNP_E_-EVs, and we have successfully prepared GMNP_E_-EVs. EVs can impact biological functions by mediating relevant biomolecules. So, we hypothesized that GMNP_E_-Evs might carry miR-15b-5p to downregulate GFAP and suppress osteogenesis. To validate the underlying molecular mechanism, we first transfected miR-15b-5p-mimic and miR-15b-5p-inhibitor separately into BMSCs. After that, EVs were extracted and loaded with GMNP_E_, and then GMNP_E_-EVs were co-cultured with transfected GFAP in RANKL-induced BMMs after 3 days.

**Fig. 7:**
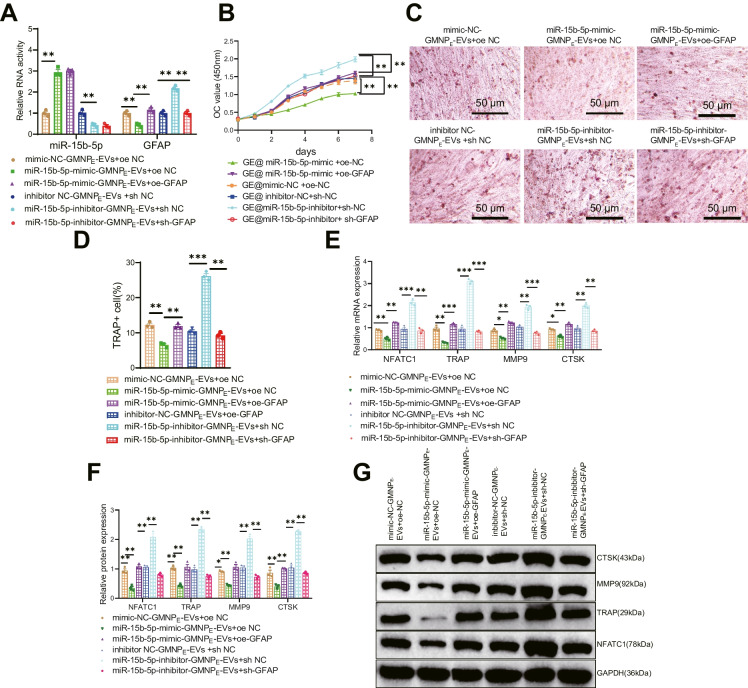
Effect of miR-15b-5p-GMNP_E_-EVs targeted inhibition of GFAP on osteoclastogenesis. Note: (**A**) Gene expression levels of GFAP and miR-15b-5p in transfected cells detected by RT-qPCR; (**B**) CCK-8 assay to assess the proliferation of osteoclasts co-cultured with GMNP_E_-EVs; (**C**-**D**) TRAP staining to assess osteoclast differentiation of BMMs after transfection (MNC ≥ 3 nuclei were considered positive); (**E**–**G**) Gene expression levels of TRAP, NFATC1, MMP9, and CTSK in transfected cells measured by RT-qPCR and Western blot. All cell experiments were repeated three times. * indicates difference between two groups with *p* < 0.05, ** indicates difference with *p* < 0.01, *** indicates difference with *p* < 0.001

RT-qPCR results showed that compared to the mimic-NC-GMNP_E_-EVs + oe-NC group, the miR-15b-5p expression level increased in the miR-15b-5p-mimic-GMNP_E_-EVs + oe-NC group, while the GFAP expression level decreased. Compared to the miR-15b-5p-mimic-GMNP_E_-EVs + oe-NC group, the GFAP expression level increased in the miR-15b-5p-mimic-GMNP_E_-EVs + oe-GFAP group. Compared to the GMNP_E_-EVs-inhibitor-NC + sh-NC group, the miR-15b-5p expression level decreased, and the GFAP expression level increased in the miR-15b-5p-inhibitor-GMNP_E_-EVs + sh-NC group. Compared to the miR-15b-5p-inhibitor-GMNP_E_-EVs + sh-NC group, the GFAP expression level decreased again in the miR-15b-5p-inhibitor-GMNP_E_-EVs + sh-GFAP group (Fig. 7A).

Subsequently, the results of detection of osteoclast proliferation and differentiation showed that compared to the mimic-NC-GMNP_E_-EVs + oe-NC group, co-culturing miR-15b-5p-mimic-GMNP_E_-EVs + oe-NC with osteoclasts resulted in a decreased osteoclast proliferation (Fig. 7B), reduced osteoclast positive cells (Fig. 7C-D), and decreased expression of osteoclast-related mRNA and proteins (Fig. 7E-G). Further overexpression of GFAP in osteoclasts was observed in the presence of miR-15b-5p-mimic-GMNP_E_-EVs, which resulted in an accelerated osteoclast proliferation (Fig. 7B), an increased number of osteoclast-positive cells (Fig. 7C-D), and an upregulation of osteoclast-related mRNA and protein expression (Fig. 7E-G), indicating a compensatory effect. Compared with the inhibitor-NC-GMNP_E_-EVs + sh-NC group, co-incubation of miR-15b-5p-inhibitor-GMNP_E_-EVs + sh-NC with osteoclasts resulted in accelerated osteoclast proliferation (Fig. 7B), increased osteoclast positivity (Fig. 7C-D), and up-regulated expression of osteoclast-related mRNA and proteins (Fig. 7E-G). Based on co-incubation with inhibitor-miRNA-15b-5p-GMNP_E_-EVs, further silencing of GFAP in osteoclasts resulted in decreased proliferation of osteoclasts (Fig. 7B), reduced osteoclast-positive cells (Fig. 7C-D), as well as decreased expression of osteoclast-related mRNA and protein (Fig. 7E-G). Therefore, miR-15b-5p-GMNP_E_-EVs were demonstrated to target GFAP and inhibit osteogenesis based on the above experiments.

MiR-15b-5p-GMNP_E_-EVs target GFAP to alleviate osteoporosis in DO rats.

Furthermore, to further validate the impact of miR-15b-5p-GMNP_E_-EVs on DO rats by regulating GFAP, we overexpressed miR-15b-5p in BMSCs cells, extracted EVs, synthesized GMNP_E_-EVs in vitro, and then injected GMNP_E_-EVs with lentiviral vectors into DO rats through the tail vein. Using an N52 neodymium magnet, an external magnetic field was applied to the femur of the rats to facilitate the enrichment of circulating GMNP_E_ nanoparticles in the femur region.

**Fig. 8:**
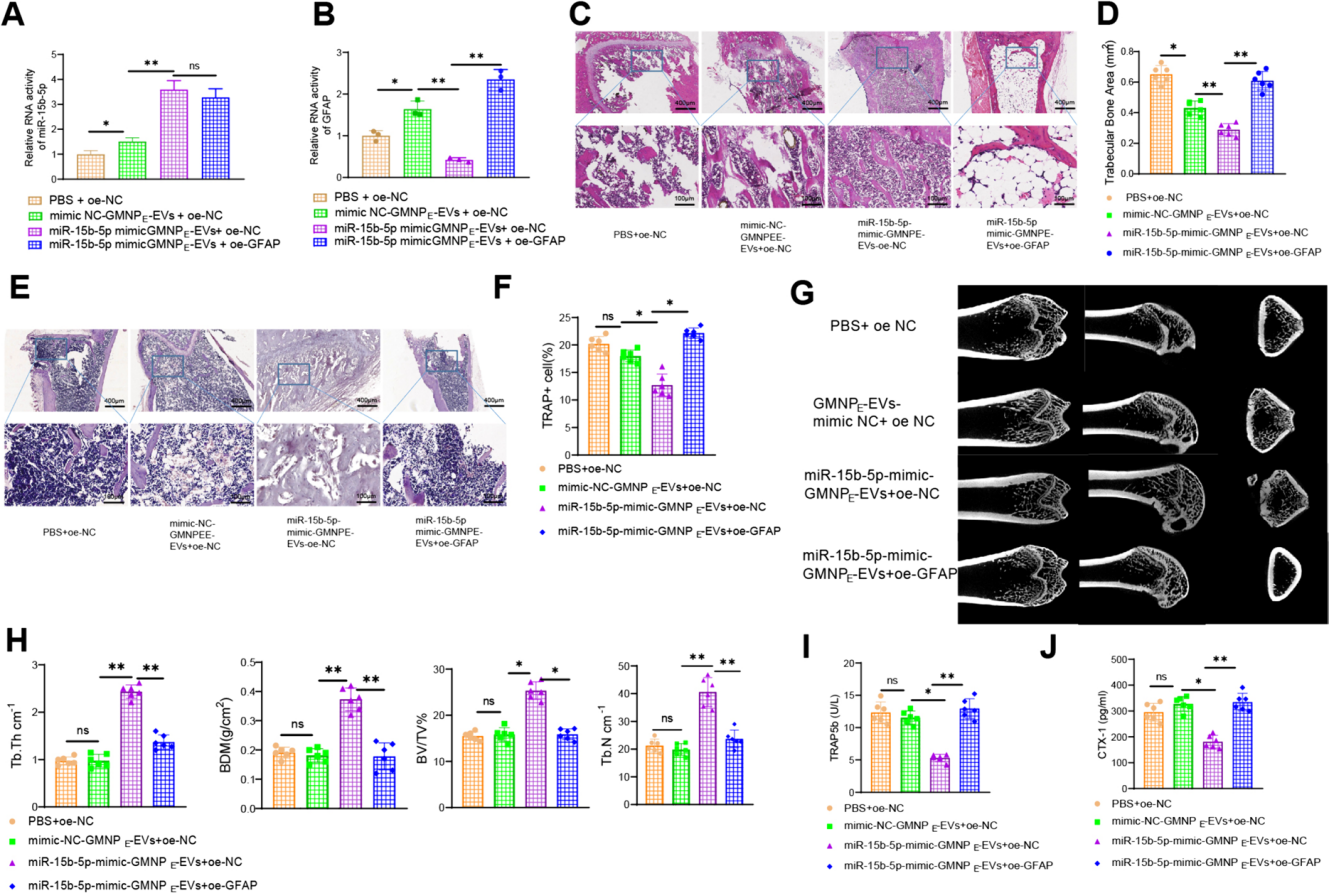
Influence of miR-15b-5p-GMNP_E_-EVs targeting GFAP in DO rats. Note: (**A**-**B**) RT-qPCR analysis of miR-15b-5p (**A**) and GFAP (**B**) expression in DO rat bone tissue; (**C**) Observation of trabecular bone area in femoral sections of DO rats using H&E staining; (**D**) Quantification of absorption area in femoral sections of DO rats from graph B; (**E**–**F**) Quantitative analysis of TRAP-positive cells in bone sections; (**G**) Micro-CT evaluation of bone formation in femoral tissue of DO rats; (**H**) Statistical analysis of BV/TV, Tb. N, Tb.Th, and BMD; (**I**-**J**) ELISA detection of serum TRAP 5b (**I**) and CTX-1 (**J**) levels in rats. Each group consisted of 6 rats. * indicates difference between two groups with *p* < 0.05, ** indicates difference with *p* < 0.01, *** indicates difference with *p* < 0.001

Firstly, the results of RT-qPCR detection showed that, compared to the PBS + oe-NC group, the mimic-NC-GMNP_E_-EVs + oe-NC group exhibited a slight increase in the expression of miR-15b-5p and a slight decrease in the expression of GFAP due to the presence of miR-15b-5p in EVs. Compared to the mimic NC-GMNP_E_-EVs + oe-NC group, the overexpression of miR-15b-5p in EVs decreased GFAP expression. Furthermore, compared to the miR-15b-5p-mimic-GMNP_E_-EVs + oe-NC group, the further overexpression of GFAP after treatment led to increased GFAP expression (Fig. 8A-B). H&E staining results showed that compared with the mimic NC-GMNP_E_-EVs + oe-NC group, the DO rat metaphyseal trabeculae in the miR-15b-5p overexpressed group were thicker, more arranged and denser, with clear and intact structures, and less depressed areas, indicating a relief of osteoporosis; compared with the miR-15b-5p-mimic-GMNP_E_-EVs + oe-NC group, the DO rat metaphyseal trabeculae in the further overexpressed GFAP-treated group were thinner, with loose structures, more significant gaps at the junctions, expanded depressed areas, and aggravated osteoporosis (Fig. 8C-D). The TRAP staining results showed that compared with the mimic-NC-GMNP_E_-EVs + oe-NC group, the number of TRAP-positive cells, which indicates osteoclast function, decreased after overexpression of miR-15b-5p in EVs. Compared with the miR-15b-5p-mimic-GMNP_E_-EVs + oe-NC group, further overexpression of GFAP increased the number of TRAP-positive cells, indicating osteoclast function protein (Fig. 8E-F).

Micro-CT examination revealed that compared to the mimic-NC-GMNP_E_-EVs + oe-NC group, overexpression of miR-15b-5p in EVs led to thicker and more compact trabeculae with a clear and intact structure at the metaphyseal end of the femur in DO rats. Additionally, the BV/TV, Tb. N, Tb. Th, and BMD values were increased, indicating an alleviation of osteoporosis. In contrast, further overexpression of GFAP after miR-15b-5p-mimic-GMNP_E_-EVs + oe-NC treatment resulted in thinner and looser trabeculae with enlarged gaps.

Moreover, the BV/TV, Tb. N, Tb. Th and BMD values were decreased, leading to a worsening of osteoporosis (Fig. 8G-H). ELISA detection results showed that, compared with the GMNP_E_-EVs- mimic NC + oe NC group, the overexpression of miR-15b-5p decreased CTX-I and TRAP 5b levels, inhibiting bone resorption and benefiting osteoporosis relief. Compared with the miR-15b-5p-mimic-GMNP_E_-EVs + oe-NC group, further overexpression of GFAP increased the levels of CTX-I and TRAP 5b in DO rats, activating bone resorption and exacerbating osteoporosis (Fig. 8I-J). It can be concluded that miR-15b-5p-GMNP_E_-EVs targeted inhibition of GFAP can alleviate osteoporosis in rats.

## Discussion

Osteoporosis is a concerning disease that has significant impacts on bone health and increases the risk of fractures for patients. However, effective treatment methods still remain a major challenge in the medical field (Sheu et al. 2023). Researchers have increasingly focused on BMSCs as a potential strategy for treating DO (Hayden et al. 2021). Previous studies have demonstrated that BMSCs play a vital role in both bone formation and remodeling (Sun et al. 2021). In recent years, researchers have closely examined EVs due to their role as key carriers of intercellular communication and their potential significance in bone health and diseases (Ren et al. 2021). Indeed, studies have shown that EVs derived from BMSCs contain a diverse array of biologically active molecules that contribute to the promotion of bone repair (Wen et al. 2021). Hence, the strategy of using BMSCs and EVs undoubtedly provides new research and treatment opportunities for DO (Xiong et al. 2021).

GFAP is a molecule that is garnering increasing attention in DO research (Brenner and Messing 2021; Chatterjee et al. 2022; Abdelhak et al. 2022). This study discovered that in the RNA-seq dataset associated with DO, the expression of GFAP was significantly elevated in the disease group, indicating its potential pivotal role in DO progression. The notable upsurge in expression is closely linked to the severity of DO, highlighting the need for a thorough comprehension of GFAP's function and regulatory mechanisms (Fu et al. 2022). Furthermore, recent findings have unveiled the crucial involvement of miR-15b-5p in this physiological process. miR-15b-5p is a proven regulatory factor that effectively targets and inhibits the expression of GFAP, thereby potentially decelerating the progression of DO. Moreover, miR-15b-5p shows a high enrichment in EVs derived from BMSCs. This finding suggests a promising therapeutic approach for regulating GFAP expression by delivering miR-15b-5p through EVs. In conclusion, by conducting an in-depth investigation into GFAP and miR-15b-5p, we not only enhance our comprehension of the molecular mechanism underlying DO, but also pave the way for novel treatment approaches (Brenner and Messing 2021).

The significant overexpression of GFAP in the bone tissue of DO rats accelerates the progression of the disease, as GFAP has the ability to enhance osteoclast differentiation. In order to identify an effective therapeutic strategy, this study utilized bioinformatics screening and in vitro cell experiments to investigate the regulatory mechanisms of GFAP. miR-15b-5p has been found to target and inhibit the expression of GFAP. However, in patients with DO, the expression of this miRNA is significantly decreased. To address this issue, the current study employs EVs derived from BMSCs loaded with GMNP_E_. These specially designed GMNP_E_ efficiently load and deliver miR-15b-5p, thereby providing it with a targeted delivery platform. In vitro experiments demonstrated that the GMNP_E_-EVs effectively delivered miR-15b-5p into osteoclasts, leading to a significant reduction in GFAP expression and further suppression of osteoclast differentiation. Furthermore, the effectiveness of this strategy has been confirmed when applied externally and internally. This study utilized GMNP_E_-EVs for the delivery of miR-15b-5p, resulting in a notable decrease in GFAP expression in the body of DO rats. This decrease in GFAP expression further inhibited osteoclast differentiation. Consequently, this method effectively alleviated the symptoms of osteoporosis in DO rats. In the research on the treatment of DO, there is a growing focus on the application of GMNP_E_ and EVs (Izquierdo et al. 2021). In this study, GMNP_E_ loaded with BMSC-derived EVs was successfully employed as a targeted delivery system for miR-15b-5p. These GMNP_E_-EVs, which have been specifically designed for this purpose, effectively deliver miR-15b-5p to the target cells, resulting in the successful regulation of GFAP. In vitro experiments have shown that this strategy can effectively inhibit the differentiation of osteoclasts. When this method was transitioned from in vitro application to in vivo, researchers observed a noteworthy reduction in osteoporosis symptoms in DO rats.

**Fig. 9:**
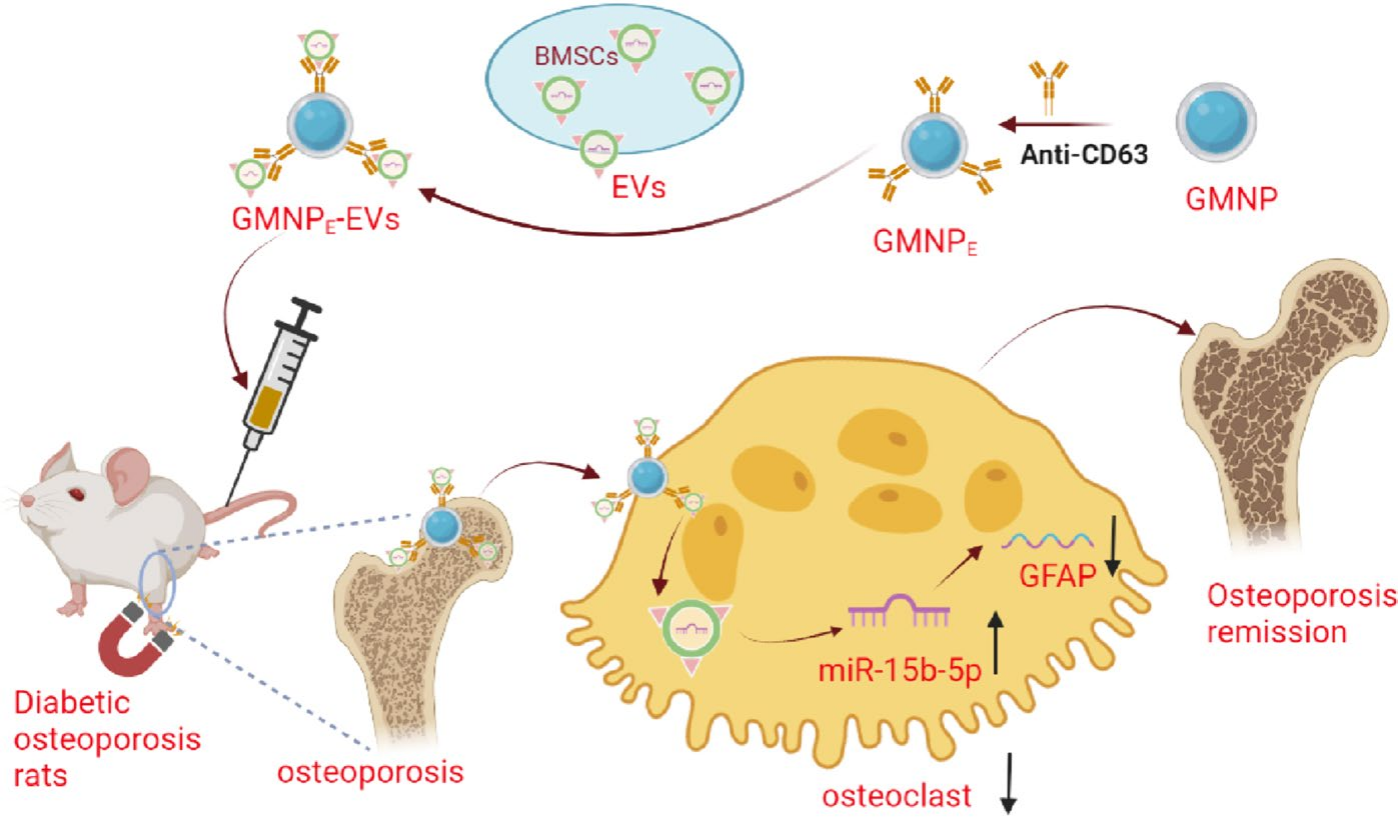
Molecular mechanism diagram of MNPs loaded with BMSCs-derived EVs delivering miR-15b-5p to regulate GFAP expression and promote osteoclastogenesis in DO progression

This study explicitly demonstrates the effectiveness of GMNP_E_-loaded BMSC-derived EVs in transferring miR-15b-5p to alleviate osteoporosis in a rat model of DO (Fig. 9). This discovery not only presents a novel molecular therapeutic mechanism but also enhances our comprehension of the molecular mechanism underlying DO. Furthermore, this therapeutic approach of combining MNPs with EVs holds great promise for the clinical treatment of DO. This could potentially offer a new treatment option for the majority of DO patients. Despite the numerous important findings yielded by this research, it is imperative to acknowledge its limitations. Further research and validation are still required to determine whether this strategy can be applied to other model animals, let alone humans.

## Supplementary Information

Below is the link to the electronic supplementary material.

Supplementary file1 (JPG 725 KB)

Supplementary file2 (JPG 528 KB)

Supplementary file3 (JPG 1.95 MB)

Supplementary file4 (JPG 1283 KB)

Supplementary file5 (DOCX 13 KB)

Supplementary file6 (DOCX 12 KB)

## Data Availability

No datasets were generated or analysed during the current study.
