# Supplementary figures and images for: Delivery of miR-15b-5p via magnetic nanoparticle-enhanced bone marrow mesenchymal stem cell-derived extracellular vesicles mitigates diabetic osteoporosis by targeting GFAP
Source: Cell Biol Toxicol. 2024 Jul 5;40(1):52. doi: 10.1007/s10565-024-09877-2 (PMC11226493; doi:10.1007/s10565-024-09877-2)

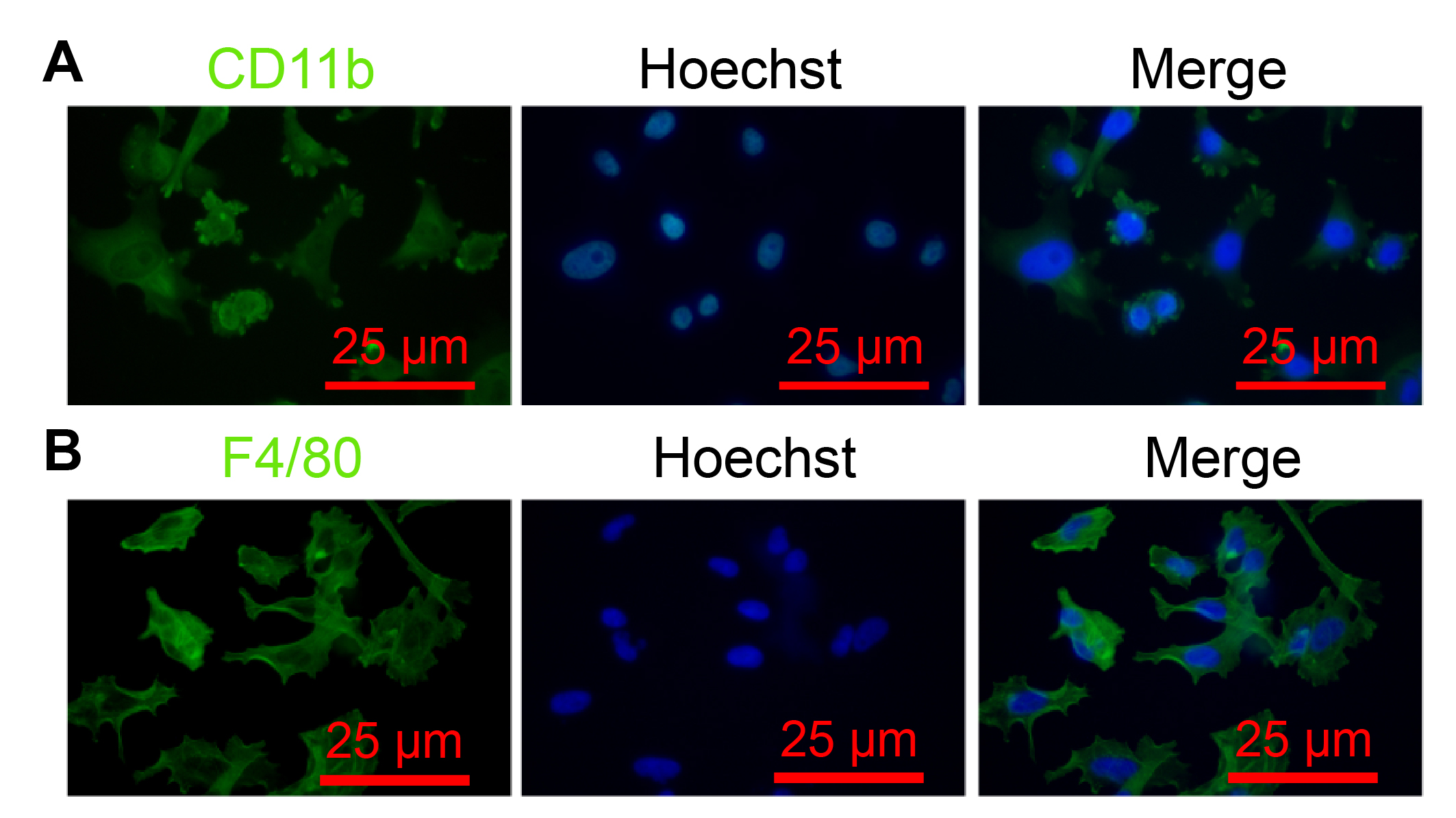

Supplement: Supplementary file 1 — Supplementary file1 (JPG 725 KB) [file 10565_2024_9877_MOESM1_ESM.jpg]

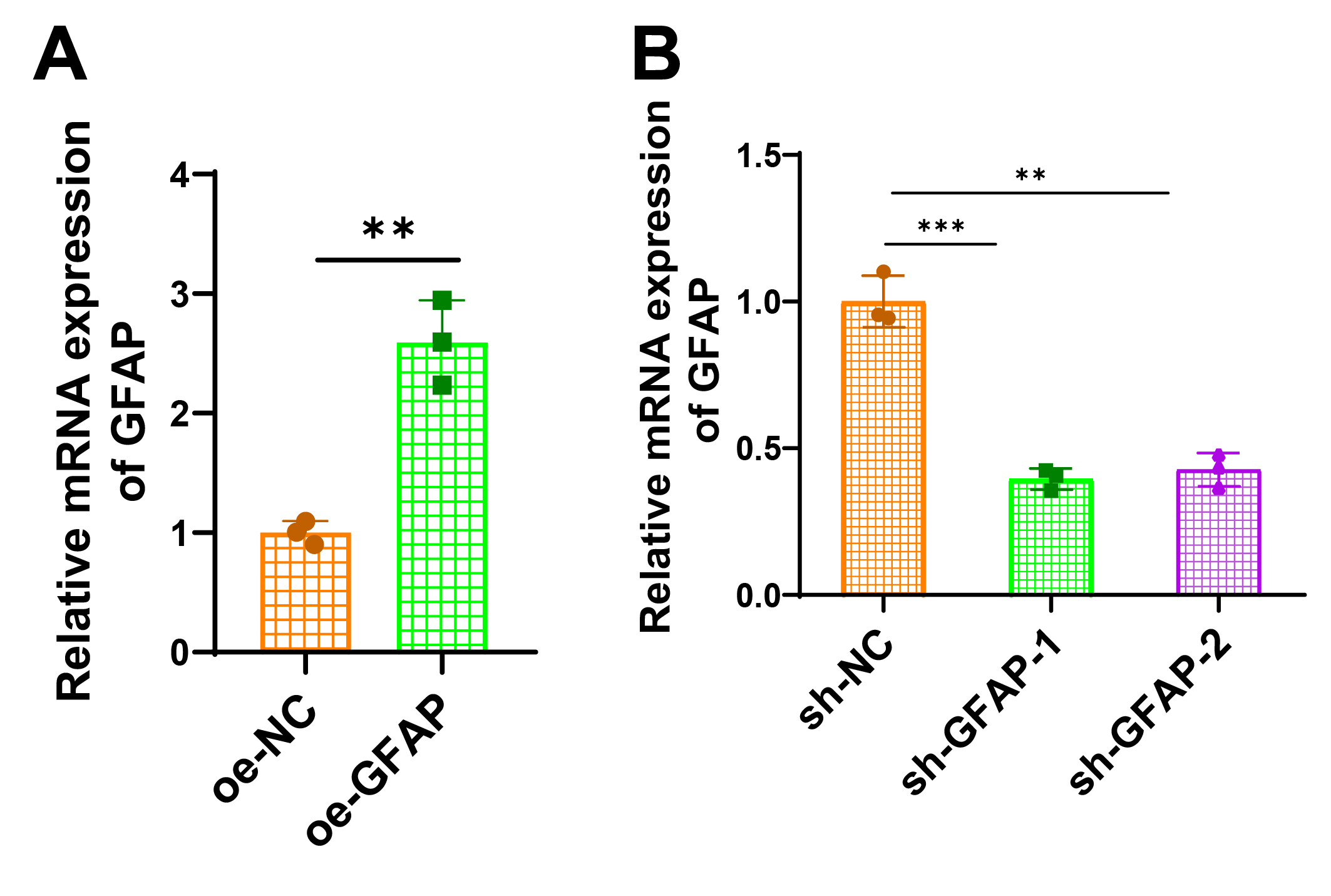

Supplement: Supplementary file 2 — Supplementary file2 (JPG 528 KB) [file 10565_2024_9877_MOESM2_ESM.jpg]

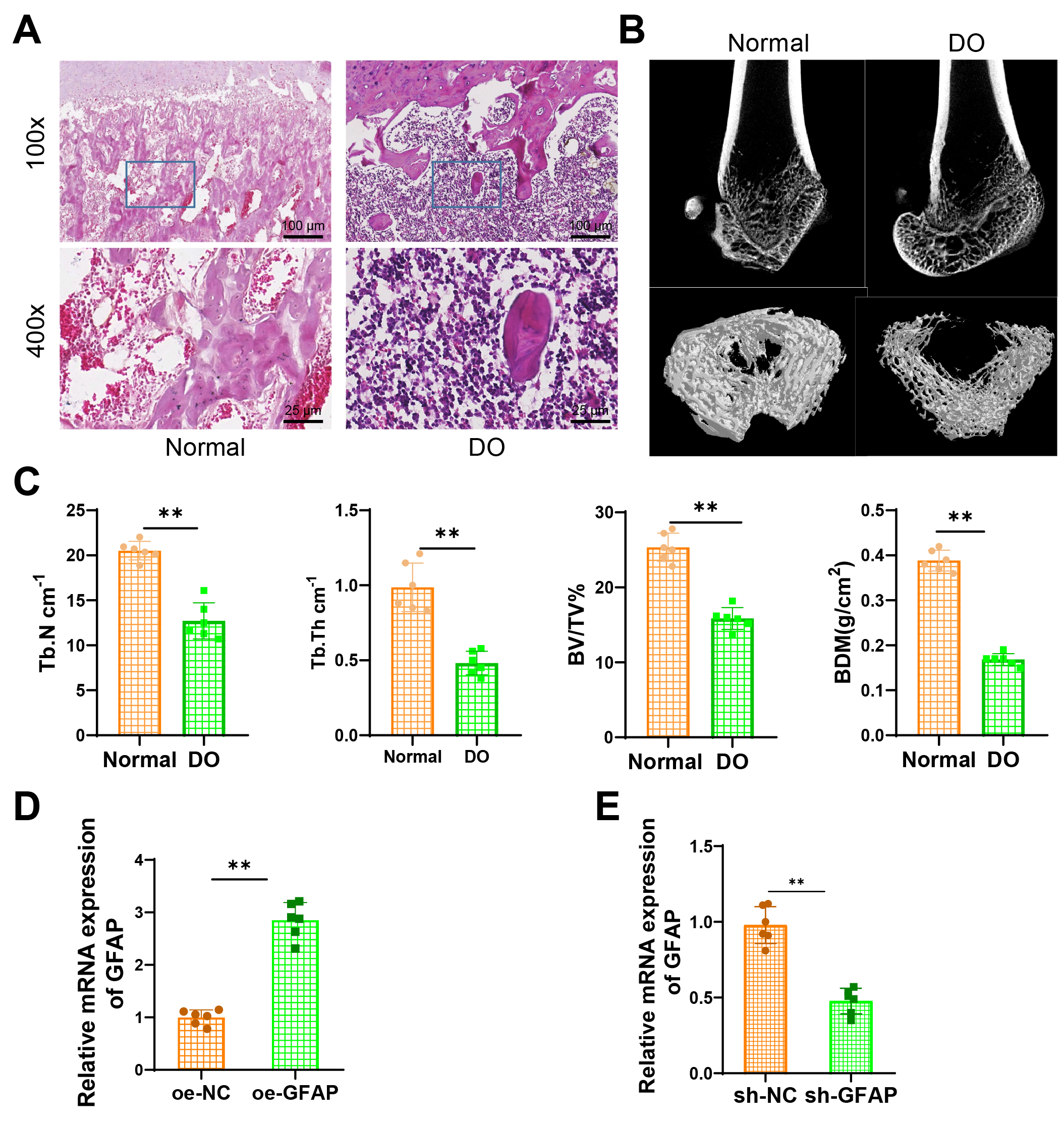

Supplement: Supplementary file 3 — Supplementary file3 (JPG 1.95 MB) [file 10565_2024_9877_MOESM3_ESM.jpg]

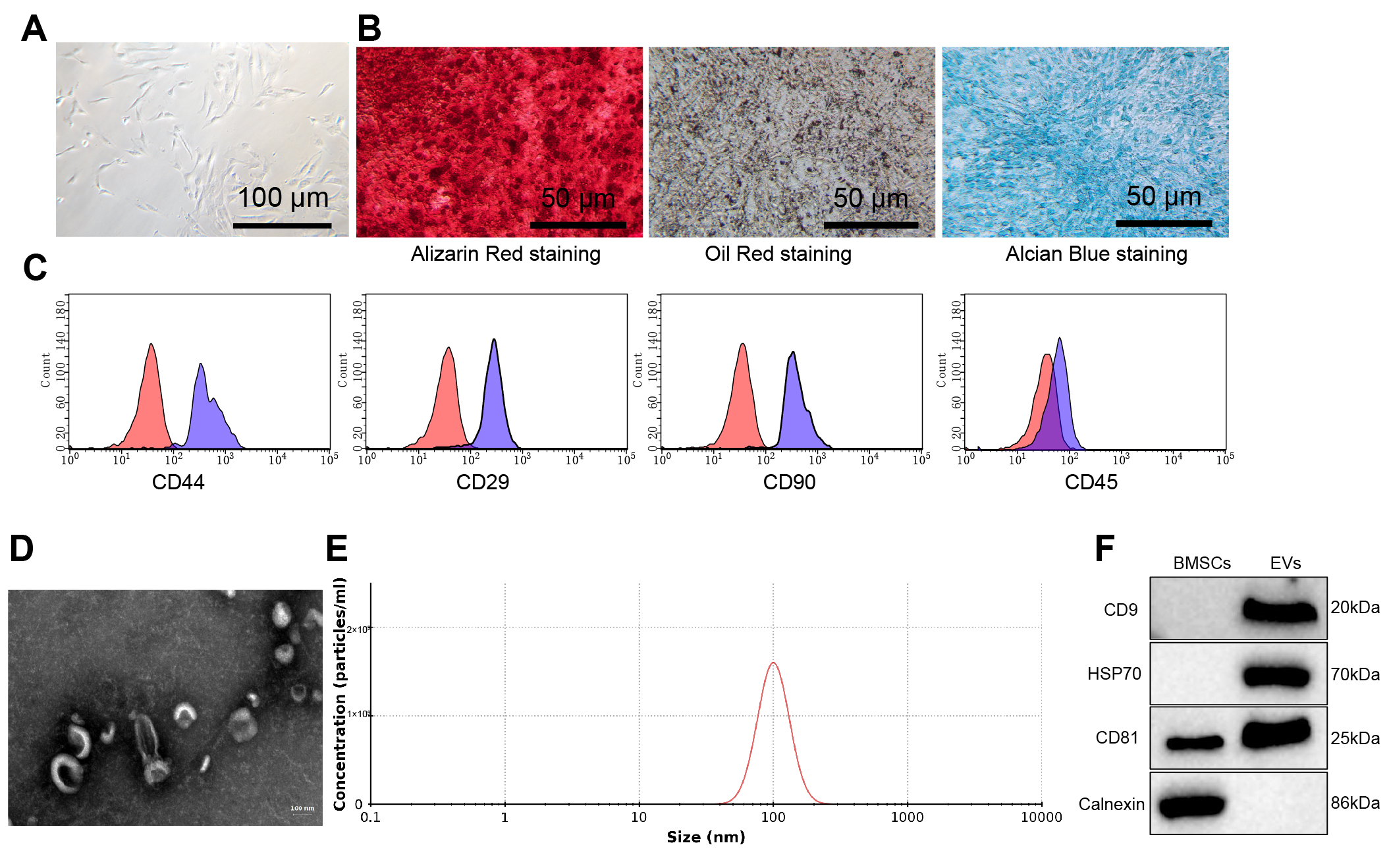

Supplement: Supplementary file 4 — Supplementary file4 (JPG 1283 KB) [file 10565_2024_9877_MOESM4_ESM.jpg]
