# Supplementary material for: Delivery of miR-15b-5p via magnetic nanoparticle-enhanced bone marrow mesenchymal stem cell-derived extracellular vesicles mitigates diabetic osteoporosis by targeting GFAP
Source: Cell Biol Toxicol. 2024 Jul 5;40(1):52. doi: 10.1007/s10565-024-09877-2 (PMC11226493; doi:10.1007/s10565-024-09877-2)
Supplement: Supplementary file 5 — Supplementary file5 (DOCX 13 KB) [file 10565_2024_9877_MOESM5_ESM.docx]

**Table S1. RT-qPCR Primer sequences**

| Gene | Primer sequences(Rat) |
| --- | --- |
| GFAP | Forward: 5'-AAATTGCTGGAGGGCGAAGA-3' |
|  | Reverse: 5'-CCGCATCTCCACCGTCTTTA-3' |
| miR-15b-5p | Forward: 5'-TAGCAGCACATCATGGTTTACA-3' |
|  | Reverse: universal reverse primer of TaqMan microRNA assay kit |
| TRAP | Forward: 5'-CACTCCCACCCTGAGATTTGT-3' |
|  | Reverse: 5'-CATCGTCTGCACGGTTCTG-3' |
| NFATC1 | Forward: 5'-GGCTACAGCCGCAGTAAATG-3' |
|  | Reverse: 5'-GCCAGCTCCAATGTGCTGAA-3' |
| CTSK | Forward: 5'-GGGAAGCAAGCACTGGATAA-3' |
|  | Reverse: 5'-GGCTGGAATCACATCTTGG-3' |
| MMP9 | Forward: 5'-GATCCCCAGAGCGTTACTCG-3' |
|  | Reverse: 5'-GTTGTGGAAACTCACACGCC-3' |
| U6 | Forward: 5'-CTCGCTTCGGCAGCACA-3' |
|  | Reverse: 5'-AACGCTTCACGAATTTGCGT-3' |
| GAPDH | Forward: 5'-AGACAGCCGCATCTTCTTGT-3' |
|  | Reverse: 5'-TACGGCCAAATCCGTTCACA-3' |
