# Supplementary material for: Delivery of miR-15b-5p via magnetic nanoparticle-enhanced bone marrow mesenchymal stem cell-derived extracellular vesicles mitigates diabetic osteoporosis by targeting GFAP
Source: Cell Biol Toxicol. 2024 Jul 5;40(1):52. doi: 10.1007/s10565-024-09877-2 (PMC11226493; doi:10.1007/s10565-024-09877-2)
Supplement: Supplementary file 6 — Supplementary file6 (DOCX 12 KB) [file 10565_2024_9877_MOESM6_ESM.docx]

**Table S2. Sequences (Invitrogen)**

| Primer | Targeted Sequences |
| --- | --- |
| sh-GFAP-1(rat) | 5’-GCTCAAT GCCGGCTTCAAAGA-3’ |
| sh-GFAP-2(rat) | 5’-GCGGAGAT GAT GGAGCTCAAT-3’ |
| sh-NC | 5’-CCTAAGGTTAAGTCGCCCTCG-3’ |
| mimic NC | 5’-UUCUCCGAACGUGUCACGUUGG-3’ |
| miR-15b-5p-mimic | 5’-TAGCAGCACATCATGGTTTACA-3’ |
| inhibitor NC | 5’-CAGUACUUUUGUGUAGUACAA-3’ |
| miR-15b-5p-inhibitor | 5’-UGUAAACCAUGAUGUGCUGCUA-3’ |
